# Human Expansion-Induced Biodiversity Crisis over Asia from 2000 to 2020

**DOI:** 10.34133/research.0226

**Published:** 2023-09-21

**Authors:** Chao Yang, Qingquan Li, Xuqing Wang, Aihong Cui, Junyi Chen, Huizeng Liu, Wei Ma, Xuanyan Dong, Tiezhu Shi, Fanyi Meng, Xiaohu Yan, Kai Ding, Guofeng Wu

**Affiliations:** ^1^ MNR Key Laboratory for Geo-Environmental Monitoring of Great Bay Area & Guangdong Key Laboratory of Urban Informatics & Shenzhen Key Laboratory of Spatial Smart Sensing and Services, Shenzhen University, Shenzhen 518060, China.; ^2^ School of Architecture and Urban Planning, Shenzhen University, Shenzhen 518060, China.; ^3^ College of Civil and Transportation Engineering, Shenzhen University, Shenzhen 518060, China.; ^4^ Guangdong Laboratory of Artificial Intelligence and Digital Economy (SZ), Shenzhen 518107, China.; ^5^ Center for Hydrogeology and Environmental Geology, China Geological Survey, Baoding 071051, China.; ^6^ Department of Geography, Hong Kong Baptist University, Hong Kong Special Administrative Region 999077, China.; ^7^ Faculty of Land Resource Engineering, Kunming University of Science and Technology, Kunming 650093, China.; ^8^ Institute for Advanced Study and Tiandu-Shenzhen University Deep Space Joint Laboratory, Shenzhen University, Shenzhen 518060, China.; ^9^ School of Civil Engineering, Chongqing Jiaotong University, Chongqing 400074, China.; ^10^ Department of Civil and Environmental Engineering, Tohoku University, Sendai 980-8579, Japan.; ^11^ School of Artificial Intelligence, Shenzhen Polytechnic, Shenzhen 518055, China.; ^12^ School of Computer Science and Technology, Dongguan University of Technology, Dongguan 523419, China.

## Abstract

Asia stands out as a priority for urgent biodiversity conservation due to its large protected areas (PAs) and threatened species. Since the 21st century, both the highlands and lowlands of Asia have been experiencing the dramatic human expansion. However, the threat degree of human expansion to biodiversity is poorly understood. Here, the threat degree of human expansion to biodiversity over 2000 to 2020 in Asia at the continental (Asia), national (48 Asian countries), and hotspot (6,502 Asian terrestrial PAs established before 2000) scales is investigated by integrating multiple large-scale data. The results show that human expansion poses widespread threat to biodiversity in Asia, especially in Southeast Asia, with Malaysia, Cambodia, and Vietnam having the largest threat degrees (∼1.5 to 1.7 times of the Asian average level). Human expansion in highlands induces higher threats to biodiversity than that in lowlands in one-third Asian countries (most Southeast Asian countries). The regions with threats to biodiversity are present in ∼75% terrestrial PAs (including 4,866 PAs in 26 countries), and human expansion in PAs triggers higher threat degrees to biodiversity than that in non-PAs. Our findings provide novel insight for the Sustainable Development Goal 15 (SDG-15 Life on Land) and suggest that human expansion in Southeast Asian countries and PAs might hinder the realization of SDG-15. To reduce the threat degree, Asian developing countries should accelerate economic transformation, and the developed countries in the world should reduce the demands for commodity trade in Southeast Asian countries (i.e., trade leading to the loss of wildlife habitats) to alleviate human expansion, especially in PAs and highlands.

## Introduction

Biodiversity is essential for sustaining food security, livelihood, ecosystem health, and economic development and for preventing future epidemics [1–5]. Biodiversity is currently declining at an unprecedented rate, and its conservation is urgent in many places [6,7]. According to the report of 7th Meeting of the International Union for Conservation of Nature (IUCN) World Conservation Congress in 2021, nearly 28% of the 138,374 species evaluated by the IUCN Red List of Threatened Species are facing extinction risk [8]. Halting biodiversity loss is the cornerstone of the Sustainable Development Goal 15 (SDG-15 Life on Land) of the United Nations’ 2030 Agenda for sustainable development [9]. The global decline of biodiversity necessitates ambitious targets for biodiversity conservation and protected area (PAs) coverage [10–13]. Currently, habitat losses and fragmentations due to intensifying human activities are major drivers of biodiversity loss [7,14–17] as human activities affect every piece of land to some degree [18]. The regions with superior natural conditions are rich in biodiversity [7,18]. However, the threats to biodiversity in these regions are often exacerbated by frequent intensive human activities as these regions are highly suitable for human developments and utilizations [14,19]. Recently, some regions or PAs with threatened species have caused special concerns because they are being greatly affected by the expansion of human activities [12,14,20,21].

Asia, with nearly 60% of the world’s population [22], stands out as a priority for urgent biodiversity conservation due to its large threatened species and PAs [8,23,24], and many countries globally are facing extreme biodiversity and ecological threats [24]. Satellite observations have shown that the human activities (i.e., cropland and artificial surface creations; for example in Fig. S1) in Asia have rapidly expanded since the 21st century and are being expanded to highlands (hilly and mountainous regions), such as the highland cropland creations in Southeast Asia [25,26] and the hillside urban land creations in China, Korea, and Malaysia [27–30]. Obviously, the intensification of human activities has induced widespread habitat losses and fragmentations and has subsequently threatened biodiversity [17,26,29,31–35]. Compared to lowlands, highlands are generally less economically and environmentally suitable for development projects [25]; however, human activities in highlands have diverged from the general view that the expansion of agricultures or human settlements in highlands is not economically feasible. Asian highlands with obvious aggregations of species play a considerable role in Earth’s biodiversity, and they are also vulnerable areas for biodiversity conservation [36–39]. Therefore, the comprehensive assessment of the threat degree of human expansion to biodiversity, especially the threat difference in lowland and highland developments, can provide a basis and new insight for biodiversity conservation and sustainable development. Unfortunately, the knowledge of threats of human expansions in lowlands and highlands to biodiversity remains unknown.

To fill this knowledge gap, a strategy is designed to address the following questions: (a) What was the threat degree of human expansion to biodiversity over 2000 to 2020 in Asia?; (b) is the threat of highland developments to biodiversity greater than that of lowland developments, in which countries and to what degree?; and (c) have the expansion regions with threats already appeared in Asian PAs? To answer these questions, the digital elevation model (DEM) and satellite-derived high-resolution land cover datasets (30-m-resolution Globeland30 datasets) are used to determine the human expansions (i.e., all cropland and artificial surface expansion patches) over 2000 to 2020 in Asian highlands and lowlands (details in Methods). Then, the IUCN Red List species and World Database of PAs (WDPA) are used to distinguish the distribution and quantity of threatened species (including mammals, amphibian, birds, reptiles, and plants) and terrestrial PAs in lowlands and highlands (details in Methods). Furthermore, a threat degree index (mainly integrating density maps of patch number, patch area, and number of threatened species involved in the patches of human expansions; Figs. S2 to S4) is proposed as a proxy for evaluating the threat degrees and differences of human expansions in highlands and lowlands to biodiversity at the continental (Asia), national (48 Asian countries), and hotspot (6,502 terrestrial PAs within Asia established before 2000) scales (details in Methods). Finally, 2 approaches are presented to assess the performance and reliability of the proposed integration index (details in Methods).

## Results

### Threat degree to biodiversity at the continental scale (Asia)

**Fig. 1.**
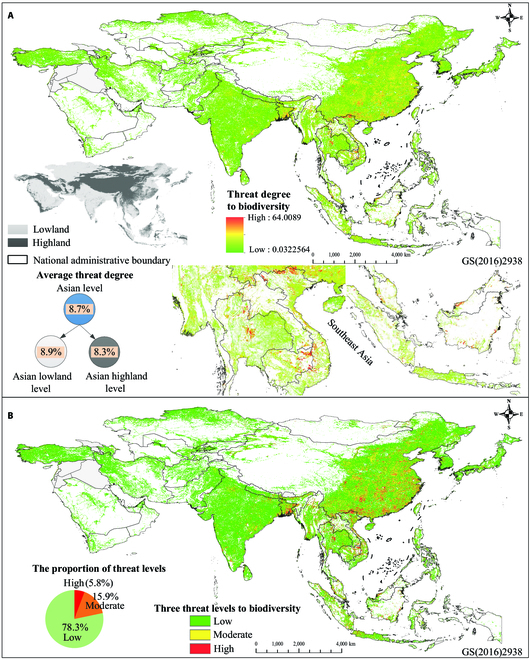
Distribution of threats of human expansion to biodiversity in Asia over 2000 to 2020. (A) Map of continuous threat degrees (1 km × 1 km cell size resolution) and average threat degrees; and (B) 3 threat levels to biodiversity (i.e., high, moderate, and low) according to the mean and standard deviation of the integration index (see Methods).

**Fig. 2.**
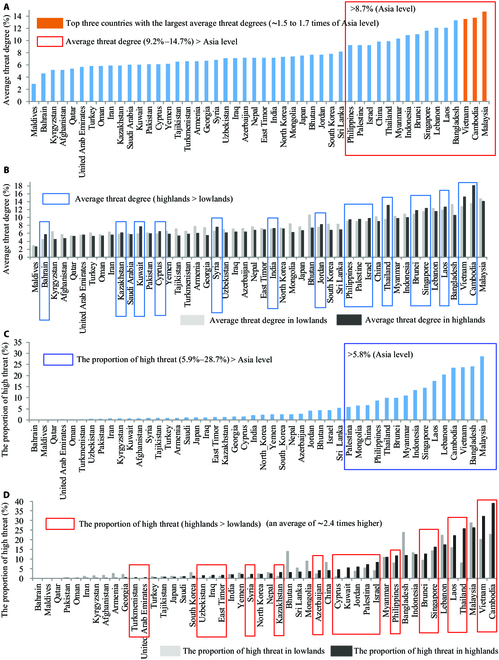
Threats of human expansion to biodiversity in the 48 Asian countries from 2000 to 2020. (A) Average threat degrees, (B) average threat degrees in lowlands and highlands, (C) proportion of high threat, and (D) proportion of high threat in lowlands and highlands.

The threat degree map of human expansion to biodiversity over Asia was generated with the proposed integration index (see Methods). The expansion of human activities over 2000 to 2020 has generally posed widespread threats to the biodiversity in Asia (Fig. 1A, wherein darker colors represent greater threat of human expansion to biodiversity). The average threat degree of human expansion to biodiversity is about 8.7% at the continental scale, and its values in the Asian highlands and lowlands are close (8.3% and 8.9%, respectively) (Fig. 1A and Table S1). The threat degree of human expansion to biodiversity in Asia was divided into 3 levels (see Methods): low, moderate, and high (Fig. 1B); and their proportions and areas were calculated (Table S2). The regions with threats (low + moderate + high) reached 8.3 million km^2^, accounting for about 26.4% of the total land area in Asia (Fig. 1B and Table S3), indicating that human expansion in Asia over 2000 to 2020 has induced an extensive threat to biodiversity. The proportions and areas of high, moderate, and low threats for entire Asia reached about 5.8% (4.8 × 10^5^ km^2^), 19.5% (1.3 × 10^6^ km^2^), and 78.3% (6.5 × 10^6^ km^2^), respectively (Fig. 2B and Table S2). In addition, moderate and low levels of threats to biodiversity have been dominant in highlands and lowlands (the sum proportion of moderate and low is >90%; Table S4). Notably, about 23% of human expansion occurred in Asian highlands during 2000 to 2020 [40]; however, the regions with threats in highlands accounted for 30.9% of the total threat areas in Asia (Table S4), suggesting that human expansions in highlands are more likely to pose a threat to biodiversity.

### Threat degree to biodiversity at national scale (48 Asian countries)

The threat degree of human expansions to biodiversity in all 48 Asian countries was investigated, and the countries exhibited great spatial heterogeneity (Fig. 1A and Table S1). In general, the average threat degrees of human expansions to biodiversity in 15 countries with average threat degrees of 9.2% to 14.7% exceeded the Asian average level (Asian average level ≈ 8.7%), and 10 of these countries belong to Southeast Asia (Fig. 2A, 15 countries inside the red border). The 3 countries with the highest average threat degrees were Malaysia (14.7%), Cambodia (13.7%), and Vietnam (13.5%), and their average threat degrees were ∼1.5 to 1.7 times that of the Asian average level (Fig. 2A), indicating that the human expansion of in these countries during 2000 to 2020 had higher impacts on biodiversity than that in other countries. The average threat degrees in the highlands exceeded the values in the lowlands in 16 countries (accounting for one-third of the Asian countries): Cambodia (18.1%), Vietnam (15.2%), Thailand (13.2%), Laos (12.7%), Singapore (12.4%), Brunei (11.8%), Israel (9.8%), Palestine (9.6%), Philippines (9.5%), Jordan (8.3%), Kuwait (7.7%), Syria (7.6%), India (7.3%), Cyprus (6.5%), Kazakhstan (6.2%), and Bahrain (5.8%) (Fig. 2B, 16 countries inside the blue border). This indicates that human expansion in highlands has induced large threats to biodiversity in one-third of the Asian countries. Notably, nearly half of the above countries are located in Southeast Asia, and their average threat degrees are much higher than those of other countries (Fig. 2B), indicating that highland development in Southeast Asian countries has posed higher threats to biodiversity than that in other Asian countries.

The proportions and areas of the 3 threat degree levels in 48 Asian countries were calculated (Supplementary Table 2). In general, China and India held the largest threatened areas (low + moderate + high), with an area of about 3.7 million km^2^ (average threat degree = 9.8%) and 1.3 million km^2^ (average threat degree = 7.2%), respectively (Fig. 1 and Tables S2 and S3). However, the proportion of threatened area of India (42.1%) is higher than China (38.9%) (Table S3), mainly due to India’s extremely high human modification [41]. The dominant threat types of most countries are moderate and low (Table S2). The proportion of high threat in 15 countries exceeded the Asian average level (Asian average level ≈ 5.8%), including all Southeast Asian countries except for East Timor, China, and Mongolia (Fig. 2C, 15 countries inside the blue border), indicating that human expansions have induced marked high threats to biodiversity in these countries, especially in Southeast Asian countries. Moreover, the proportions of high threat areas in the highlands were larger than those in lowlands in 20 Asian countries (an average of ∼2.4 times higher) (Fig. 2D, 20 countries inside the red border), indicating that the highland developments in these country have induced larger high threats to biodiversity than lowland developments. Cambodia (39.0%), Vietnam (32.4%), Malaysia (26.3%), Thailand (25.9%), and Laos (22.3%) exhibited the largest proportions of high threat areas in highlands [∼5.3 to 9.3 times of the Asian average level (Asian average level ≈ 4.2%)] (Table S4).

### Threat degree to biodiversity at the hotspot scale (6,502 PAs within Asia)

**Fig. 3.**
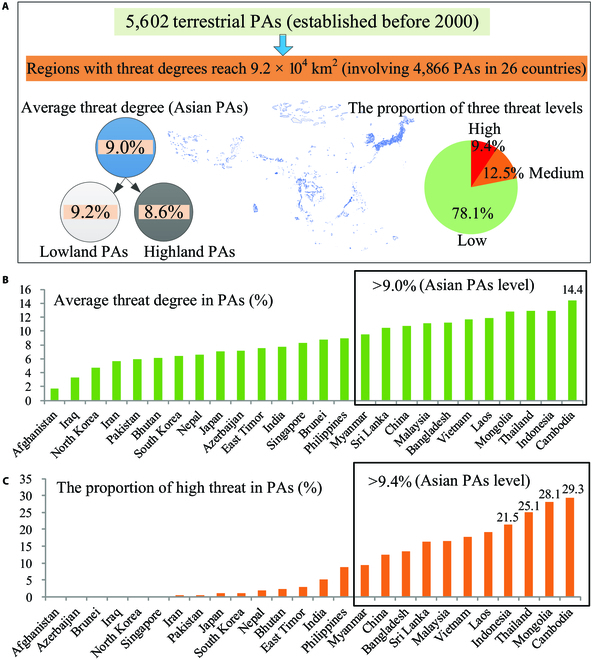
Threats of human expansion to biodiversity in Asian PAs from 2000 to 2020. (A) Average threat degrees and proportions of threat types in all Asian PAs, (B) average threat degrees in 26 countries (only 26 countries’ PAs exhibited threat degree regions in Asia), and (C) proportions of high threats in 26 countries.

Totally, 6,502 terrestrial PAs established before 2000 have been set up in Asia (excluding point and after merging the overlapped features), and their total area is about 9.8 ×10^5^ km^2^, with highland PAs and lowland PAs accounting for 56.8% and 43.2% of the total area, respectively (Table S5). According to the observed threat degrees of human expansion to biodiversity in Asian PAs, regions with threat degrees have emerged in ∼75% Asian PAs (including 4,866 PAs in 26 countries), and their total area is about 9.2 × 10^4^ km^2^ (accounting for ∼9.4% of the total area of Asian PAs) (Fig. 3A and Table S3). The average threat degree of Asian PAs has reached 9.0%, which is higher than the Asian average level (Asian average level ≈ 8.7%) (Fig. 3A and Table S1), indicating that human expansion in PAs has induced greater threats to biodiversity than that in non-PAs. Notably, the average threat degree in the PAs of 11 countries was >9.0% (Asian PAs average level), including Cambodia, Indonesia, Thailand, Mongolia, Laos, Bangladesh, Malaysia, Vietnam, Sri Lanka, China, and Myanmar, and the PAs of Cambodia exhibited the largest average threat degree (reaching 14.4%) (Fig. 3B). This suggests that the effectiveness of these countries in controlling human expansion in the PAs over 2000 to 2020 was lower than that of other Asian countries, especially for Cambodia. Moreover, the average threat degrees of PAs in highlands in Vietnam, Thailand, and Myanmar were markedly higher than that in lowlands (Table S6).

The proportions and areas of high, moderate, and low threats in all Asian PAs reached about 9.4% (8.7 × 10^3^ km^2^), 12.5% (1.1 × 10^4^ km^2^), and 78.1% (7.2 × 10^4^ km^2^), respectively (Fig. 3A and Table S2). The proportion of high threats in all PAs was markedly higher than the Asian average level (Asian average level ≈ 5.8%) (Fig. 3A and Table S2), indicating that human expansion in PAs induced considerably high threats to biodiversity than that in non-PAs (approximately 1.6 times). The proportions of high threats in the PAs of 10 countries exceeded the average level of Asian PAs (~9.4%), and the proportions for Cambodia, Mongolia, Thailand, and Indonesia were greater than 20% (Fig. 3C and Table S7), indicating that human expansion in PAs in these countries (especially for Cambodia, Mongolia, Thailand, and Indonesia) had greater high threats to biodiversity than that in other countries over 2000 to 2020. The proportions of high threats in both lowland and highland PAs in Cambodia exceeded 25% (Table S7), indicating that Cambodia’s lowland and highland PAs were strongly disturbed by human expansion.

## Discussion

This study obtained new insight on the threats of human expansion to biodiversity over Asian lowlands and highlands in the 21st century at the continental, national, and hotspot scales. The transparent spatiotemporal characteristics of the threat degrees obtained herein can provide foundations to governments, ecologists, and biodiversity conservation organizations for regulating human expansion for biodiversity conservation. Moreover, the threat degrees to biodiversity in lowlands and highlands provide urgent insights for the future sustainable developments of human beings. The integration index for assessing the threat degrees proposed herein can be visualized in temporally and spatially explicit ways and can be easily transferred to other study areas. Two strategies were applied to confirm the performance and reliability of the proposed integration index (see Method). First, human activities on lands, especially the expansion of croplands and artificial surfaces, directly result in habitat loss and strong interference to biodiversity [42–45], and, thus, the accuracy of human expansion mainly determines the reliability of our results. The Globeland30-derived human expansion dataset used herein was assessed in our previous study [40] with more than 10,000 high-resolution (≤5 m) samples, and the overall agreement was greater than 90%, verifying that the reliability of our results (see Methods) as an overall agreement >85% denotes high accuracy [46,47]. Spatially, the accuracy of detecting human expansions in the highlands has been very similar to that in the lowlands [40], suggesting that the difference of the threat degree results between highlands and lowlands is reliable. Second, the average correlation degree reflects the comprehensive representation ability and suitability of the composite index [48,49]. The correlation of the threat degree was tested against 3 density maps (patch number, patch area, and number of threatened species involved in patches of human expansions). The results show that the average correlation degree of the integration index with the 3 density maps (∼0.53, *P* < 0.01) is far higher than the average correlation degree of the single indicators (Table S9), suggesting that our proposed integration index exhibits stronger comprehensive representation and suitability than the single indicators and it can comprehensively reflect the threats of human expansions to biodiversity,

Biodiversity can enhance the ecosystem functioning [50], and, thus, a higher threat to biodiversity from human expansions might weaken the ecosystem functioning. Since the average threat degree of 15 countries exceeded the Asian average level, most of which (10 countries) belong to Southeast Asia, the ecosystem functions of these countries declined to a greater extent than those of other countries, especially in Southeast Asian countries. These findings confirm the reasons why Southeast Asia has become an urgent global hotspot region in biodiversity conservation [24–26,31]. Previous studies proved that the demands of timber, cash crops (corn), tea, coffee, and upland rice intensified human expansions (e.g., agriculture, logging, and artificial surface) in Southeast Asian countries [40,51–54], and these expansions have widely extended to the highlands in the 21st century [25,40]. Since Southeast Asia has abundant tropical forests and rich species [24,25,32,54], the human expansions easily pose threats to biodiversity [6,14,15,55,56], and, thus, Southeast Asian countries exhibit larger threat degrees than other countries; in particular, Malaysia, Cambodia, and Vietnam exhibit the highest average threat degrees. Moreover, intensified economic activities (e.g., goods purchase and consumption) in biodiversity richness areas can induce threats for wildlife [57]. Southeast Asia has become the hotspot of species threats due to commodity trade (i.e., trade leading to the loss of wildlife habitat) from the European Union and the United States [57], which verifies the high threat degrees in Southeast Asian countries observed in this study.

The results also show that over 2000 to 2020, highland developments posed higher threats to biodiversity than lowland developments in one-third Asian countries (half of which are Southeast Asian countries), while they induced large proportions of high threat areas in 20 countries (90% of which are Southeast Asian countries). These results could be explained by that the following reasons: Because of terrain constraints, highland developments have higher economic costs than lowland developments [58], leading to more scattered and fragmented expansion patches (Fig. S1), while the threatened species in highlands are richness in abovementioned Asian countries (even more than those in lowlands) (Table S10), and, thus, highland developments easily involve more threatened species, especially in Southeast Asian countries (Fig. S4C). These highland developments will greatly hinder SDG-15 compared to lowland developments in many Asian countries, especially in Southeast Asian countries. Therefore, to achieve SDG-15, the Asian countries with higher threat degrees in highlands should accelerate economic transformation to reduce excessive dependence on cropland and artificial surface creations in highlands and realize intensive developments in lowlands.

Some areas with high threat degrees (Fig. 1) may also increase the possibility of human–animal conflicts and decrease the biodiversity in Asia, especially in highlands or PAs. Recently, the conflicts between humans and endangered animals have been observed in China (Yunnan and Sanjiangyuan Reserve) and Indonesia (Aceh) [59–63], such as human conflicts with Asian elephants (*Elephas maximus* and *Sumatran elephant*) [61–63] and bears (*Ursus arctos pruinosus*) [59,60]. Conflict areas have been observed in the high threat areas obtained herein, further supporting the reliability of our results. Frequent conflicts have mainly been observed because of the replacement or erosion of a species’ habitat by human expansions [61,63], especially in highlands. The personal injury and property loss caused by wildlife often deteriorate the relationship between humans and animals, prompting the retaliatory killings of the threatened species [60].Therefore, the supervision of high threat areas could reduce the occurrence of these conflicts and better protect biodiversity. Terrestrial PAs worldwide are frequently set up in remote and high-altitude areas with less human activities [64,65]. However, the regions with threats to biodiversity have been observed in ∼79% Asian terrestrial PAs in the 21st century, and the average threat degrees in these regions are far higher than the Asian average level. Human expansions in PAs are likely to induce higher threats because PAs comprise more threatened species (both animal and plats) than other regions. Since PAs are the hotspots and cornerstones of biodiversity conservation [66], their supervision needs to be strengthened.

To our knowledge, this study is the first to reveal the threats of human expansion to biodiversity in Asia at multiple scales over 2000 to 2020. Considering the widespread threats of human expansions to biodiversity in Asia, especially in Southeast Asian countries, all countries need to regulate biodiversity by reducing commodity trade (i.e., trade leading to the loss of wildlife habitat), similar to how some countries are now responding to carbon emissions. Subsequently, human expansions could be limited to a certain extent (especially highland agriculture expansions), and the threat to biodiversity could be reduced. Furthermore, Asian developing countries could accelerate economic transformation to reduce highland developments, especially in Southeast Asian countries. The developed countries (e.g., European Union countries and the United States) should reduce the demands for commodity trade in Southeast Asian countries to alleviate human expansion, especially to highlands and PAs.

## Methods

The threats of human expansions to biodiversity in lowlands and highlands were revealed at 3 scales: continental (Asia), national (48 Asian countries), and hotspot (6,502 terrestrial PAs within Asia established before 2000) scales. First, the lowlands and highlands were mapped, and all human expansion patches (mainly referring to cropland and artificial surface expansion patches over 2000 to 2020) in Asia were detected. Then, the number of threatened species and their distribution regions (e.g., terrestrial mammals, amphibian, birds, reptiles, and plants) and the terrestrial PAs in Asian lowlands and highlands were quantified. Finally, a threat degree index was developed by fusing the density maps of patch number, patch area, and number of threatened species involved in the patches, and the threat degrees and differences of human expansions in lowlands and highlands to biodiversity were evaluated at the continental, national, and hotspot scales. The performance and reliability of the proposed threat degree index were also evaluated using different strategies. All spatial datasets processing and analyses were performed in World Geodetic System 1984 coordinate system and Albers Equal Area Conic projection.

### Mapping Asian lowlands and highlands

The Advanced Spaceborne Thermal Emission and Reflection Radiometer Global Digital Elevation Model (ASTER GDEM ) (3,935 DEM tiles) dataset of Asia with a 30-m spatial resolution, which were captured around and after 2000, was downloaded from the National Aeronautics and Space Administration (NASA) website (https://search.earthdata.nasa.gov/search/). ASTER GDEM was jointly developed by the Ministry of Economy Trade and Industry of Japan and NASA of the United States by stacking all individual cloud- and noncloud-masked scene DEMs while applying various algorithms to remove the abnormal data [67]. ASTER GDEM products have been widely used in various fields, and their reliability has been proven [68]. DEM was used to derive the lowlands and highlands of Asia (Fig. 1A, bottom corner image) according to the elevation and slope class criteria afforded by Margono et al. [69]. Highlands include hills (elevation of 0 to 1,000 m with a slope of >15°, elevation of 1,000 to 1,200 m with a slope of >8°, or elevation of 1,200 to 1,500 with a slope of 0° to 3°) and mountains (elevation of 1,200 to 1,500 with a slope of >3° or elevation of >1,500 m); lowlands refer to plains and terraces (elevation of 0 to 1,000 m with a slope of 0° to 15° or elevation of 1,000 to 1,200 m with a slope of 0° of 8°) (Table S11).

### Detecting human expansion patches

Since a small human expansion patch may affect biodiversity [7,18], especially for land creations in highlands, satellite-derived high-resolution land cover products of 2000 and 2020 (30-m-resolution Globeland30 datasets; http://www.globallandcover.com/) were used to detect human expansion patches. Globeland30 datasets were developed by combining several multispectral data sources, including Landsat Thematic Mapper, Enhanced Thematic Mapper Plus, Chinese HJ-1 images, and Gaofen multispectral images (2020 version), and they have been extensively used for various areas [70]. The classification was performed using a split-and-merge strategy, Pixel–Object–Knowledge approach was applied to classify each land cover type, and a knowledge-based interactive verification procedure was applied to improve the mapping accuracy [71,72]. The accuracy of the Globeland30 dataset was assessed using the landscape shape index-based sampling model and more than 230,000 samples, and the overall accuracy and kappa coefficient, respectively, were 85.7% and 0.82 for the 2020 version and about 83.5% and 0.78 for the previous versions [72], which meet the accuracy requirements of land cover change analysis, especially for large-scale applications [46,47]. Cropland and artificial surface expansions were selected as human expansion representatives because they cover almost all intensive human activities (such as agriculture, industry, transportation, and human settlement expansions) from the definition of land cover types of Globeland30 [40]. The human expansions over 2000 to 2020 in Asia were determined using the change detection method (i.e., the pixels were labeled as human expansions if they were croplands or artificial surfaces in 2020 but not in 2000). To count the number and area of patches, the human expansion pixels were converted to vector data using 8-neighborhood rule in ArcGIS 10.4 platform. These expanding patches represent a summary of highland and lowland developments (i.e., land creations) in Asia from 2000 to 2020.

### Identifying threatened species and distribution regions

We extracted the IUCN Red List species (mammals, amphibians, reptiles, birds, and plants) for the Asian region from the current global species distribution data [73,74]. These species have been the most comprehensively evaluated, and, thus, polygon maps (vector file) are available. The distribution data of terrestrial vertebrate (terrestrial mammal, amphibian, bird, and reptile) and plant species were obtained from the IUCN Red List database [73] (version 6.2, updated on January 2019; https://www.iucnredlist.org), while data on bird species were obtained from the Birdlife International website [74] (version 2020.1; http://datazone.birdlife.org/home). Threatened species comprised the Critically Endangered (CR), Endangered (EN), and Vulnerable (VU) Red List categories [2,8]. The attribute values of scientific name (binomial) and protection priority (i.e., category of CR, EN, and VU) were combined to determine all threatened species (CR + EN + VU) and their distribution boundaries in Asia, including mammals (438 species), amphibians (503 species), reptiles (351 species), birds (442 species), and plants (269 species) (Table S10). The overlap method was used to derive the number of threatened species in lowlands and highlands. The total number of threatened species (CR + EN + VU) reached 2003 (i.e., mammal + amphibian + reptiles + birds + plants) over Asia (mainly refers to 3,819 spatial layers), with 1,985 and 1,837 species in highlands and lowlands, respectively (Table S10).

### Identifying terrestrial PAs

The entire Asian and Pacific PA dataset was collected from the WDPA [23] (https://www.protectedplanet.net/en; acquired in December 2021) afforded by the United Nations Environment Programme’s World Conservation Monitoring Centre, and all terrestrial PA distribution boundaries in Asia were refined. To avoid overestimating the human expansion in PAs (as some PAs could be established after human expansion), we excluded PAs established after 2000 in our study. When the status year of PAs is not available, we also excluded these records. After excluding the point features and merging overlapped features, 6,502 terrestrial PAs established before 2000 (mainly refers to 11,258 polygon features) in Asia were obtained on the basis of the name field of PAs, comprising an area of about 9.8 × 10^5^ km^2^. Finally, the areas of lowland and highland PAs were calculated (highland PAs accounting for 56.8% and lowland PAs accounting for 43.2%) (Table S5).

### Assessing the threat degrees of human expansions to biodiversity

Since the interference of human expansions on threatened species is more likely to directly or indirectly lead to the decline and extinction of species and ultimately pose great threats to biodiversity [2,14,16,33,34,75,76], this study developed a threat degree index (Eq. 1) as a proxy for measuring the threat degrees of human expansions to biodiversity. Theoretically, higher patch number of human expansions indicates higher fragmentation and lower landscape connectivity of threatened species regions [17,33,76]. A larger patch size of human expansions generally results in more serious habitat loss of threatened species regions [42,76–79]. Moreover, a larger number of threatened species involved in the expansion patches (defined as Fig. S2) denotes a higher threat to biodiversity [14,42,76,77]. Therefore, an integration index was proposed herein on the basis of the above indicators as follows. To produce connected grid images, 1 km × 1 km grid cells were generated using the Asian boundary, and the intersection analysis method and traversal algorithm were used to identify the patch number, patch area, and the number of threatened species involved in the patches in every grid (calculated as Fig. S3) by combining the Asian grid cells, distributions of human expansion patches, and threatened species. After traversing all grid cells, the density maps of patch number, patch area, and threatened species involved in the patches were obtained (Fig. S4). Finally, the threat degree index was calculated as the geometric mean of these 3 indicators [80]:

$${\mathrm{TD}}_{i}=\sqrt[{3}]{\Delta N_{i}\times \Delta A_{i}\times \Delta T_{i}}$$ (1)

where *TD_i_* represents the threat degree of human expansion to biodiversity in time period *i*, Δ*N_i_* is the normalized density map of the patch number, Δ*A_i_* represents the normalized density map of the patch area, and Δ*T_i_* is the normalized density map of the threatened species. Specifically, Δ*N_i_*, Δ*A_i_*, and Δ*T_i_* were calculated using the normalized formula (Eq. 2) and were dimensionless. The *TD_i_* value varied from 0% to 100%, with a larger value indicating a higher threat of human expansion to biodiversity.

$$\Delta X=\left(X-X_{\min}\right)/\left(X_{\max}-X_{\min}\right)\times 100\%$$ (2)

where Δ*X* represents the normalized density map of *X* (i.e., Δ*N_i_*, Δ*A_i_*, and Δ*T_i_*) and *X_min_* and *X_min_* are the minimum and maximum values in the image of *X*, respectively.

The integration index was used to derive the map of the threat degrees of human expansion to biodiversity over Asia (Fig. 1A). The average values of the threat degree at the continental (Asia), national (48 Asian countries), and hotspot (PAs within Asia) scales were calculated for analysis (including the overall threat degree, highland-threat degree, and lowland-threat degree, see columns 2 to 3 in Supplementary Table 1). The threat degree map of Asia was classified into 3 levels according to the mean and standard deviation of the integration index [81]: low, moderate, and high (Table).Then, the proportions and areas of the 3 threat levels were obtained (Fig. 1B and Table S2). The difference values of the average threat degrees in highlands and lowlands in Asia were determined using Eq. 3 to explore the threat difference in highlands and lowlands (see last column in Table S1).

$$D_{i}=\text{Highland}\left({\mathrm{TD}}_{i}\right)-\text{Lowland}\left({\mathrm{TD}}_{i}\right)$$ (3)

**Table.** Classification of threat degree map of Asia.

Threat types	Low	Moderate	High
Dividing criteria	<$\bar{x}$ + 0.5*s*	$\bar{x}$ + 0.5*s* to $\bar{x}$ + 1.5*s*	>$\bar{x}$ + 1.5*s*
Threat degree index	<~11.74	11.74–17.82	>17.82

Note: $\bar{x}$ and *s* represent the mean and standard deviation of threat degree index, respectively.

where *D_i_* represents the difference values of the threat degrees of human expansion to biodiversity in highlands and lowlands and *Highland*(*TD_i_*) and *Lowland*(*TD_i_*) represent the threat degrees in highlands and lowlands, respectively.

### Evaluating integration index

Two strategies were used to evaluate the performance and reliability of our proposed integration index on the threat degrees of human expansion to biodiversity. First, the human activities on lands, especially the expansion of croplands and artificial surfaces, directly affect the habitats of species and cause strong interference to biodiversity [42–45], and, thus, the accuracy of human expansion detection mainly determines the accuracy of our results. The overall accuracy of the Globeland30-derived human expansions (i.e., cropland and artificial land expansions) was assessed by a third party [40] with more than 10,000 high-resolution (≤5 m) samples. The overall agreement of human expansion was more than 90%, and the accuracy of human expansion in the highlands was very similar to that in the lowlands (about 93% versus 94%) [40]. Second, the average correlation degree reflects the comprehensive representation ability and suitability of the integration index [48], and a higher average correlation degree between the integration index and each component, compared to the average correlation between components, indicates a stronger comprehensive representativeness and suitability of the integration index [49]. Therefore, the correlation of the proposed integration index against 3 density maps (patch number, patch area, and number of threatened species involved in patches) was investigated. Specifically, 3,074 samples were randomly generated using random function from the threat degree map (samples were evenly distributed across Asia with an interval of 3 × 3 km), and the locations (longitude and latitude) and pixel values (threat degree) of these sample points were obtained (see Supplementary Data). Then, the longitude and latitude of these samples were used to obtain the pixel values of the 3 density maps at the same position (see Supplementary Data). Finally, correlation (Pearson) and significance tests were conducted, and the average correlation degree of the integration index with the density maps was obtained (Table S9). The significance test and correlation analysis were performed using SPSS Statistics 19.

### Uncertainties and limitations

Some uncertainties and limitations still need to be explored. First, this study used cropland and artificial surface creations as representative of human expansion but did not consider some potential rare human expansions (e.g., hunting and trapping). Second, we used artificial impervious surface and cropland to represent the threat of human activities on PA, and previous investigations have shown that the human influences mean more artificial impervious surface and cropland [41,82]. However, the road could be a determinant variable in evaluating the effectiveness of PA and biological conservation. Whether the different representations of human activities could bring more bias into the evaluation of human pressure on biological conservation has not been evaluated in our study; therefore, more exploration is needed in future work. Third, we calculated the threat degree index as the geometric mean of the density maps of patch number, patch size, and number of threatened species involved in the patches. Although the threat degree index can effectively evaluate the impact of human expansion to biodiversity, it may hold uncertainty in a certain special situation. For example, more patches and larger area of human expansion may have fewer threated species, and fewer patches and smaller area of human expansion may have more threated species, the form of consecutive multiplication in the threat degree index could weaken the differences between these 2 cases. In addition, the threat degree was classified on the basis of the mean and standard deviation of integration index. Other common classification approaches need to further explore, such as Jenks natural break, equal interval, and quantile. Finally, the IUCN Red List was used as representative of threatened species in Asia, and it includes some unknown threatened species, which may cause the underestimation of the threat degree. Although the PAs provided by WDPA were considered, different countries may have some local PAs, which have not been evaluated by WDPA, and future studies can further assess the threats to local PAs.

## Data Availability

The DEM dataset is available in NASA (https://search.earthdata.nasa.gov/search/). The high-resolution land cover products (Globeland30) are available in the National Geomatics Center of China (http://www.globallandcover.com/). Threatened species (mammals, amphibians, reptiles, and plant) were obtained from IUCN Red List database (https://www.iucnredlist.org/). Threatened species (birds) were obtained from Birdlife International website (http://datazone.birdlife.org/home). The Asian PAs data are available in the WDPA (http://www.wdpa.org/). The data that support the findings are all publicly available online (see Results and the Supplementary Materials). The raster data of threat degree map of Asia are available in a public data repository (https://zenodo.org/record/8181386). No custom codes were used. All map-related operations were performed using ArcGIS (10.4) platform (www.esri.com/en-us/arcgis). The statistical analysis was performed using SPSS Statistics (19) (www.ibm.com/cn-zh/spss). The processing program and equations are illustrated in Methods.

## Supplementary Materials

Figs. S1 to S4 Tables S1 to S11 Supplementary Data (Excel)
