# Supplementary material for: Human Expansion-Induced Biodiversity Crisis over Asia from 2000 to 2020
Source: Research (Wash D C). 2023 Sep 21;6:0226. doi: 10.34133/research.0226 (PMC10513745; doi:10.34133/research.0226)
Supplement: Supplementary file 1 — Figs. S1 to S4 Tables S1 to S11 Supplementary Data (Excel) [file research.0226.f1.docx]

Supplementary Materials for

**Human Expansion-Induced Biodiversity Crisis over Asia from 2000 to 2020**

**Chao Yang ^1, 2^, Qingquan Li ^1, 3,4^** ^*^**, Xuqing Wang ^5^, Aihong Cui ^6^, Junyi Chen^7^ , Huizeng Liu ^1,8^, Wei Ma ^9^, Xuanyan Dong ^10^, Tiezhu Shi ^1, 2^, Fanyi Meng^1, 3^,** **Xiaohu Yan ^11^, Kai Ding ^12^, Guofeng Wu ^1, 2^**

1. MNR Key Laboratory for Geo-Environmental Monitoring of Great Bay Area & Guangdong Key Laboratory of Urban Informatics & Shenzhen Key Laboratory of Spatial Smart Sensing and Services, Shenzhen University, Shenzhen 518060, China;

2. School of Architecture and Urban Planning, Shenzhen University, Shenzhen 518060, China;

3. College of civil and transportation engineering, Shenzhen University, Shenzhen 518060, China;

4. Guangdong Laboratory of Artificial Intelligence and Digital Economy (SZ), Shenzhen 518107, China;

5. Center for Hydrogeology and Environmental Geology, China Geological Survey, Baoding 071051, China;

6.Department of Geography, Hong Kong Baptist University, Hong Kong Special Administrative Region 999077, China;

7. Faculty of Land Resource Engineering, Kunming University of Science and Technology, Kunming 650093, China;;

8. Institute for Advanced Study & Tiandu-Shenzhen University Deep Space Joint Laboratory, Shenzhen University, Shenzhen 518060, China;

9. School of Civil Engineering, Chongqing Jiaotong University, Chongqing 400074, China;

10. Department of Civil and Environmental Engineering, Tohoku University, Sendai 980-8579, Japan;

11. School of Artificial Intelligence, Shenzhen Polytechnic, Shenzhen 518055, China;

12. School of Computer Science and Technology, Dongguan University of Technology, Dongguan 523419, China

**Materials include:**

**Supplementary Figs.1-4; Supplementary Tables 1-11**

**Supplementary Fig.1. Typical human expansions in Asian highlands (left: cropland creation, right: artificial surface creation).The images were obtained from Google Earth Pro®.**

**
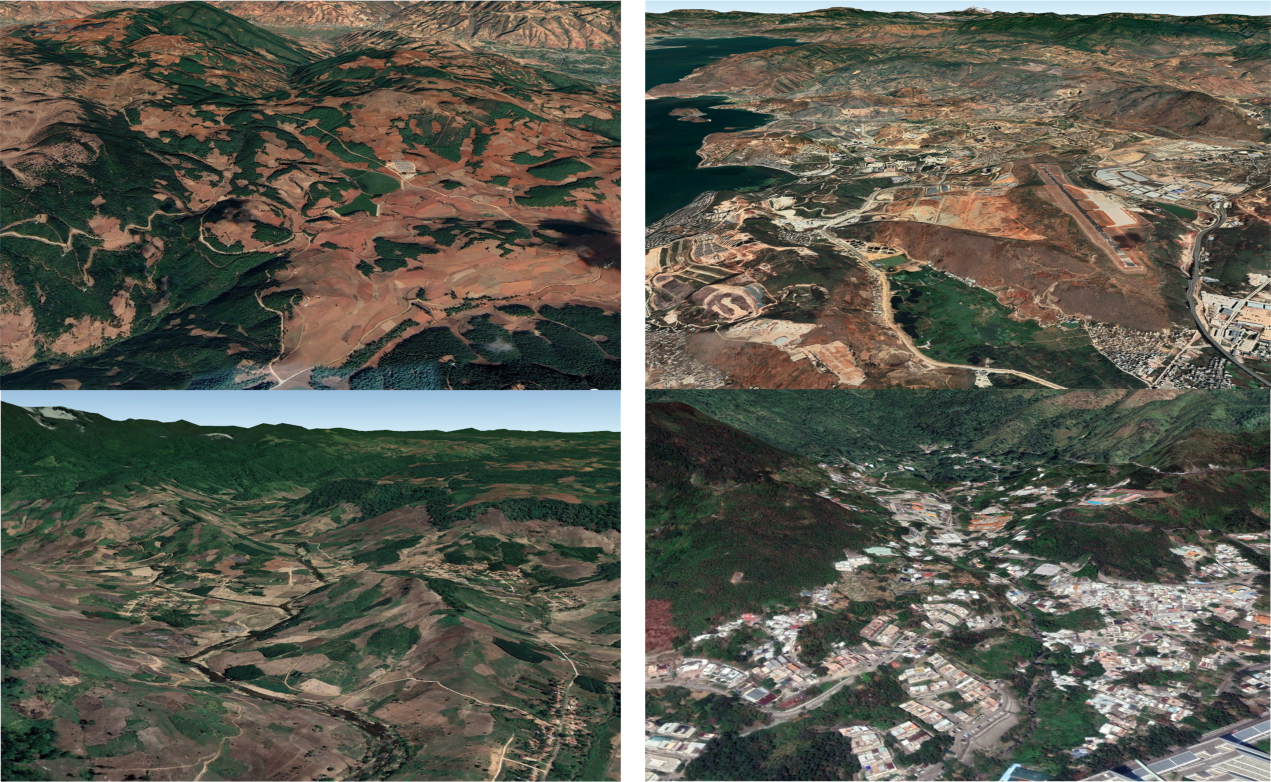
**

**Supplementary Fig.2.** **The principle for calculating the number of threatened species involved in the human expansion patches.**


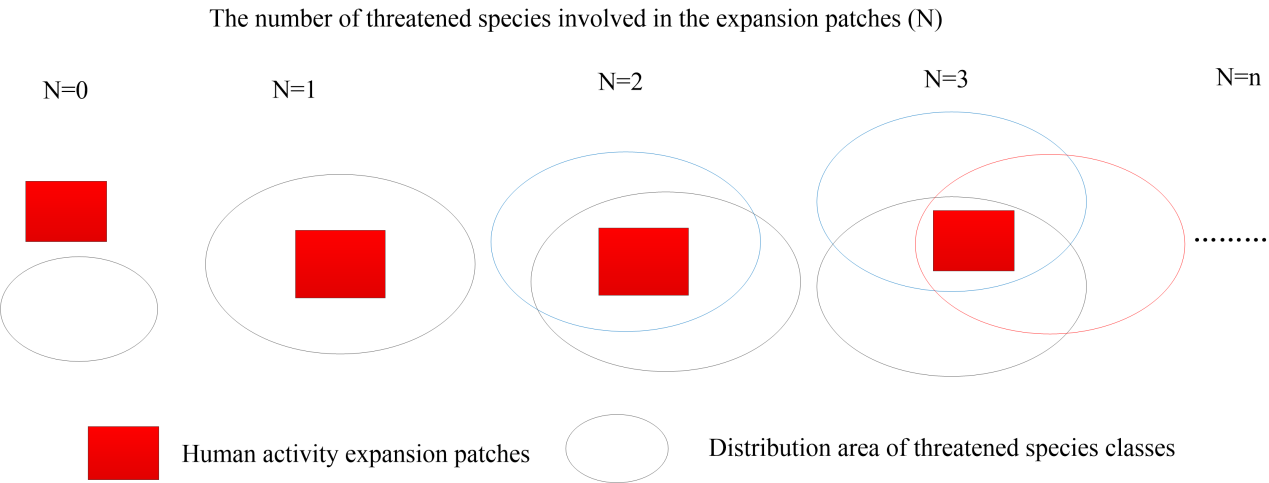


**Supplementary Fig.3. The principle for calculating the three parameters of integration index ( i.e., threat degree index). (A) calculation of density map of patch number, (B) calculation of density map of patch area, and (C) calculation of density maps of threatened species involved in patches.**


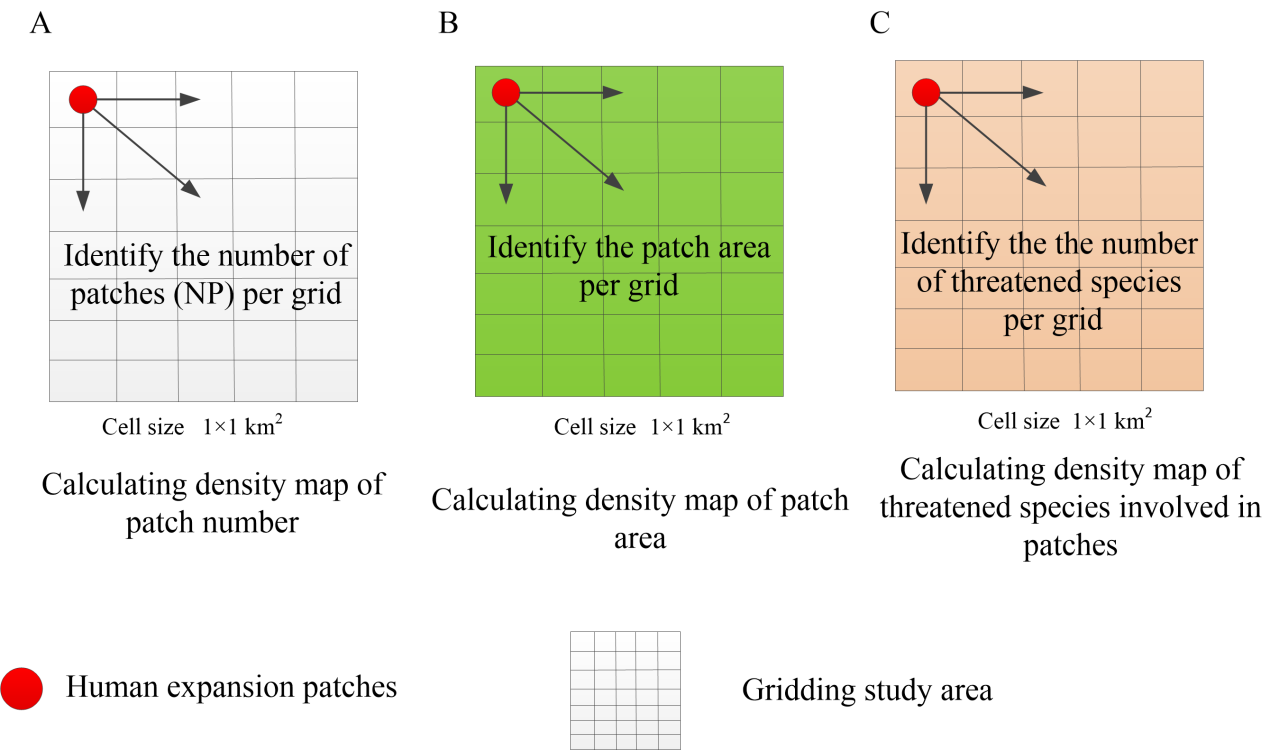


**Supplementary Fig.4. The density maps of three parameters of developed integration index in our study. (A) density map of patch number of human expansions, (B) density map of patch area of human expansions, and (C) threatened species involved in patches of human expansion.**


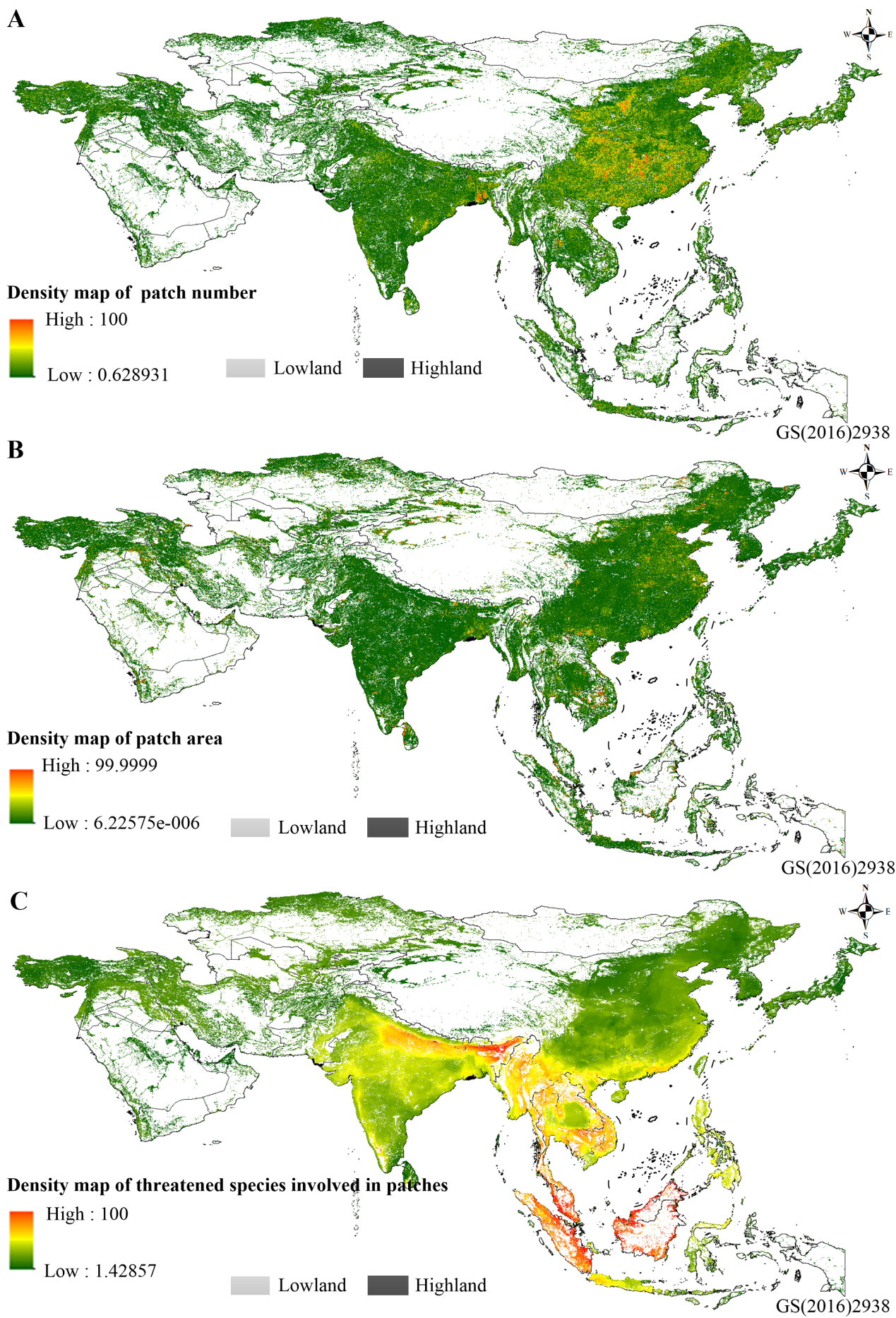


**Supplementary Table 1. The average values of threat degrees in continental (Asia), national (48 Asian countries) and hotspot (6502 terrestrial PAs within Asia) scale.**

| **Continental scale** | Overall average  (%) | Lowland average  (%) | Highland average  (%) | Difference value (highland-lowland) (%) |
| --- | --- | --- | --- | --- |
| Asia | 8.7 | 8.9 | 8.3 | -0.6 |
| **National scale** |  |  |  |  |
| Afghanistan | 5.2 | 5.8 | 4.8 | -1.0 |
| Armenia | 6.6 | 7.9 | 6.1 | -1.8 |
| Azerbaijan | 7.2 | 7.3 | 6.4 | -0.9 |
| Bahrain | 4.6 | 4.6 | 5.8 | 1.2 |
| Bangladesh | 13.3 | 13.4 | 10.6 | -2.8 |
| Bhutan | 7.7 | 10.7 | 7.4 | -3.3 |
| Brunei | 11.0 | 10.9 | 11.8 | 0.9 |
| Cambodia | 13.7 | 13.6 | 18.1 | 4.5 |
| China | 9.8 | 10.3 | 9.1 | -1.3 |
| Cyprus | 6.1 | 6.0 | 6.5 | 0.5 |
| East Timor | 7.2 | 7.3 | 7.0 | -0.3 |
| Georgia | 6.7 | 7.5 | 5.5 | -1.9 |
| India | 7.2 | 7.2 | 7.3 | 0.1 |
| Indonesia | 10.9 | 11.0 | 10.1 | -0.9 |
| Iran | 5.9 | 6.4 | 5.7 | -0.7 |
| Iraq | 7.1 | 7.2 | 6.4 | -0.8 |
| Israel | 9.2 | 9.1 | 9.8 | 0.7 |
| Japan | 7.5 | 7.7 | 6.8 | -1.0 |
| Jordan | 7.7 | 7.6 | 8.3 | 0.7 |
| Kazakhstan | 5.9 | 5.9 | 6.2 | 0.3 |
| Kuwait | 6.0 | 6.0 | 7.7 | 1.7 |
| Kyrgyzstan | 5.2 | 6.5 | 4.6 | -1.9 |
| Laos | 12.1 | 11.9 | 12.7 | 0.8 |
| Lebanon | 12.1 | 12.4 | 11.6 | -0.9 |
| Malaysia | 14.7 | 14.8 | 14.2 | -0.7 |
| Maldives | 2.9 | 3.0 | 2.7 | -0.3 |
| Mongolia | 7.4 | 8.5 | 6.3 | -2.2 |
| Myanmar | 10.3 | 10.4 | 9.9 | -0.5 |
| Nepal | 7.2 | 7.8 | 6.9 | -0.8 |
| North Korea | 7.3 | 7.4 | 7.2 | -0.2 |
| Oman | 5.8 | 5.8 | 5.4 | -0.4 |
| Pakistan | 6.1 | 6.2 | 6.0 | -0.1 |
| Palestine | 9.2 | 9.1 | 9.6 | 0.4 |
| Philippines | 9.2 | 9.2 | 9.5 | 0.3 |
| Qatar | 5.4 | 5.4 | 5.3 | -0.1 |
| Saudi Arabia | 6.0 | 6.0 | 5.8 | -0.1 |
| Singapore | 11.6 | 11.6 | 12.4 | 0.8 |
| South Korea | 7.8 | 8.4 | 6.9 | -1.5 |
| Sri Lanka | 8.2 | 8.4 | 7.0 | -1.3 |
| Syria | 6.8 | 6.7 | 7.6 | 0.9 |
| Tajikistan | 6.5 | 7.2 | 5.4 | -1.8 |
| Thailand | 9.9 | 9.6 | 13.2 | 3.6 |
| Turkey | 5.8 | 6.2 | 5.3 | -0.8 |
| Turkmenistan | 6.6 | 6.6 | 5.9 | -0.7 |
| United Arab Emirates | 5.7 | 5.7 | 5.7 | 0.0 |
| Uzbekistan | 7.1 | 7.1 | 6.2 | -0.9 |
| Vietnam | 13.5 | 12.8 | 15.2 | 2.4 |
| Yemen | 6.2 | 6.6 | 5.9 | -0.7 |
| **Hotspot scale** |  |  |  |  |
| Asian terrestrial PAs | 9.0 | 9.2 | 8.6 | -0.6 |

**Supplementary Table 2. Proportions (%) and areas (km^2^) of three threat degrees (i.e., high, moderate and low) at continental (Asia), national (48 Asian countries) and hotspot ( 6502 terrestrial PAs within Asia) scale.**

| **Continental scale** | High threat | | Moderate threat | | Low threat | |
| --- | --- | --- | --- | --- | --- | --- |
| Asia | 475214.5 | 5.8% | 1317461.2 | 15.9% | 6470290.2 | 78.3% |
| **National scale** |  |  |  |  |  |  |
| Afghanistan | 752.8 | 0.8% | 2232.6 | 2.4 % | 89973.6 | 96.8% |
| Armenia | 87.4 | 1.0% | 507.8 | 5.6 % | 8512.7 | 93.5% |
| Azerbaijan | 848.6 | 2.8% | 2601.5 | 8.6 % | 26880.0 | 88.6% |
| Bahrain | 0.0 | 0.0% | 2.5 | 0.9 % | 270.7 | 99.1% |
| Bangladesh | 23800.0 | 24.0% | 29114.0 | 29.4 % | 46127.7 | 46.6% |
| Bhutan | 232.3 | 4.3% | 586.8 | 10.9 % | 4556.3 | 84.8% |
| Brunei | 79.5 | 10.0% | 205.4 | 25.9 % | 509.1 | 64.1% |
| Cambodia | 14125.9 | 23.5% | 13538.9 | 22.5 % | 32381.3 | 53.9 % |
| China | 242640.7 | 6.6 % | 839128.0 | 22.7 % | 2613524.8 | 70.7 % |
| Cyprus | 67.5 | 1.6 % | 309.8 | 7.3 % | 3895.7 | 91.2% |
| East Timor | 22.7 | 1.3 % | 210.5 | 12.0 % | 1515.5 | 86.7 % |
| Georgia | 342.7 | 1.5 % | 2013.4 | 8.7 % | 20889.8 | 89.9% |
| India | 28442.6 | 2.2 % | 117962.0 | 9.1 % | 1153553.6 | 88.7 % |
| Indonesia | 37641.4 | 13.4 % | 53012.6 | 18.8 % | 191258.0 | 67.8 % |
| Iran | 1926.5 | 0.7 % | 11603.0 | 4.0 % | 273831.4 | 95.3 % |
| Iraq | 832.9 | 1.2 % | 6471.6 | 9.0 % | 64375.3 | 89.8 % |
| Israel | 358.3 | 4.4 % | 1657.8 | 20.2 % | 6188.9 | 75.4 % |
| Japan | 1917.3 | 1.1 % | 19299.3 | 11.4 % | 147530.9 | 87.4 % |
| Jordan | 390.2 | 4.2 % | 1091.8 | 11.8 % | 7738.3 | 83.9 % |
| Kazakhstan | 4918.2 | 1.3 % | 17371.0 | 4.7 % | 351126.3 | 94.0 % |
| Kuwait | 18.0 | 0.7 % | 84.3 | 3.4 % | 2355.2 | 95.8 % |
| Kyrgyzstan | 149.1 | 0.7 % | 689.3 | 3.1 % | 21191.1 | 96.2 % |
| Laos | 8323.2 | 17.5 % | 10503.4 | 22.1 % | 28672.8 | 60.4 % |
| Lebanon | 1162.4 | 20.5 % | 1431.4 | 25.2 % | 3088.9 | 54.4 % |
| Malaysia | 14551.1 | 28.7 % | 10600.1 | 20.9 % | 25537.9 | 50.4 % |
| Maldives | 0.0 | 0.0 % | 0.0 | 0.0 % | 50.7 | 100.0 % |
| Mongolia | 2042.5 | 6.5 % | 2286.5 | 7.3 % | 26917.5 | 86.1 % |
| Myanmar | 20007.1 | 11.0 % | 35949.2 | 19.7 % | 126310.3 | 69.3 % |
| Nepal | 1531.1 | 2.6 % | 4122.2 | 7.0 % | 53350.3 | 90.4 % |
| North_Korea | 938.6 | 2.4 % | 4046.7 | 10.4 % | 33820.5 | 87.2 % |
| Oman | 20.5 | 0.2 % | 437.3 | 4.1 % | 10145.8 | 95.7 % |
| Pakistan | 913.0 | 0.5 % | 10030.7 | 5.6 % | 166801.5 | 93.8 % |
| Palestina | 180.7 | 5.9 % | 623.6 | 20.2 % | 2280.2 | 73.9 % |
| Philippines | 6149.5 | 8.8 % | 10043.2 | 14.3 % | 53919.7 | 76.9 % |
| Qatar | 0.0 | 0.0 % | 22.2 | 1.0 % | 2313.1 | 99.0 % |
| Saudi Arabia | 941.2 | 1.1 % | 3891.9 | 4.4 % | 83130.3 | 94.5 % |
| Singapore | 70.0 | 14.5 % | 127.0 | 26.2 % | 287.1 | 59.3 % |
| South_Korea | 1732.7 | 2.6 % | 8385.2 | 12.5 % | 57224.4 | 85.0 % |
| Sri_Lanka | 1671.3 | 5.4 % | 4323.4 | 14.0 % | 24913.4 | 80.6 % |
| Syria | 395.9 | 0.9 % | 3058.5 | 6.8 % | 41514.1 | 92.3 % |
| Tajikistan | 140.0 | 0.9 % | 897.5 | 5.7 % | 14842.5 | 93.5 % |
| Thailand | 18763.7 | 9.9 % | 33395.8 | 17.6 % | 138123.5 | 72.6 % |
| Turkey | 2973.8 | 0.9 % | 14711.7 | 4.6 % | 303830.2 | 94.5 % |
| Turkmenistan | 83.8 | 0.3 % | 1635.8 | 4.9 % | 31432.4 | 94.8 % |
| United Arab Emirates | 22.0 | 0.2 % | 405.7 | 3.3 % | 11716.7 | 96.5 % |
| Uzbekistan | 331.9 | 0.5 % | 5041.7 | 7.5 % | 61851.7 | 92.0 % |
| Vietnam | 31929.9 | 23.6 % | 30374.5 | 22.5 % | 72741.5 | 53.9 % |
| Yemen | 743.8 | 2.5 % | 1421.8 | 4.8 % | 27287.2 | 92.6 % |
| **Hotspot scale** |  |  |  |  |  |  |
| Asian terrestrial PAs | 8653.6 | 9.4% | 11490.3 | 12.5% | 71683.4 | 78.1% |

**Supplementary Table 3. Total threat area (km2) and Proportions (%) of three threat degrees (i.e., high, moderate and low) at continental (Asia), national (48 Asian countries) and hotspot (6502 terrestrial PAs within Asia) scale.**

| **Continental scale** | Land area (administrative boundary area) | Threat area (low+ moderate+ high) | Proportions (Threat area/ Land area) |
| --- | --- | --- | --- |
| Asia | 31249599.0 | 8262965.9 | 26.4% |
| **National scale** |  |  |  |
| Afghanistan | 643857.5 | 92959.0 | 14.4% |
| Armenia | 29690.2 | 9107.9 | 30.7% |
| Azerbaijan | 86235.5 | 30330.1 | 35.2% |
| Bahrain | 715.1 | 273.2 | 38.2% |
| Bangladesh | 139384.4 | 99041.7 | 71.1% |
| Bhutan | 37710.5 | 5375.4 | 14.3% |
| Brunei | 5777.8 | 794.0 | 13.7% |
| Cambodia | 181359.6 | 60046.1 | 33.1% |
| China | 9488601.6 | 3695293.5 | 38.9% |
| Cyprus | 9268.1 | 4273.1 | 46.1% |
| East Timor | 14915.5 | 1748.7 | 11.7% |
| Georgia | 69844.6 | 23245.8 | 33.3% |
| India | 3085092.4 | 1299958.2 | 42.1% |
| Indonesia | 1890244.8 | 281911.9 | 14.9% |
| Iran | 1621741.7 | 287360.9 | 17.7% |
| Iraq | 445407.8 | 71679.8 | 16.1% |
| Israel | 22158.9 | 8204.9 | 37.0% |
| Japan | 372468.1 | 168747.5 | 45.3% |
| Jordan | 89116.9 | 9220.3 | 10.3% |
| Kazakhstan | 2730537.3 | 373415.5 | 13.7% |
| Kuwait | 17388.8 | 2457.5 | 14.1% |
| Kyrgyzstan | 198649.3 | 22029.5 | 11.1% |
| Laos | 230001.6 | 47499.4 | 20.7% |
| Lebanon | 10238.1 | 5682.7 | 55.5% |
| Malaysia | 329383.4 | 50689.0 | 15.4% |
| Maldives | 299.7 | 50.7 | 16.9% |
| Mongolia | 1566250.9 | 31246.5 | 2.0% |
| Myanmar | 669297.4 | 182266.6 | 27.2% |
| Nepal | 147662.7 | 59003.6 | 40.0% |
| North_Korea | 122755.1 | 38805.8 | 31.6% |
| Oman | 309336.6 | 10603.6 | 3.4% |
| Pakistan | 874177.3 | 177745.2 | 20.3% |
| Palestina | 6220.0 | 3084.5 | 49.6% |
| Philippines | 295856.8 | 70112.4 | 23.7% |
| Qatar | 11625.0 | 2335.3 | 20.1% |
| Saudi | 1915940.6 | 87963.4 | 4.6% |
| Singapore | 697.0 | 484.1 | 69.5% |
| South_Korea | 100229.2 | 67342.3 | 67.2% |
| Sri_Lanka | 65837.4 | 30908.1 | 46.9% |
| Syria | 186918.4 | 44968.5 | 24.1% |
| Tajikistan | 142113.1 | 15880.0 | 11.2% |
| Thailand | 514054.6 | 190283.0 | 37.0% |
| Turkey | 780704.5 | 321515.6 | 41.2% |
| Turkmenistan | 489592.8 | 33152.1 | 6.8% |
| United Arab Emirates | 71206.0 | 12144.4 | 17.1% |
| Uzbekistan | 447554.4 | 67225.3 | 15.0% |
| Vietnam | 329276.3 | 135046.0 | 41.0% |
| Yemen | 452203.6 | 29452.8 | 6.5% |
| **Hotspot scale** |  |  |  |
| Asian terrestrial PAs | 975549.0 | 91827.2 | 9.4% |

**Supplementary Table 4. Proportions (%) and areas (km^2^) of three threat degrees (i.e., high, moderate and low) in lowlands and highlands at continental (Asia), national (48 Asian countries) and hotspot (6502 terrestrial PAs within Asia) scale.**

| **Continental scale** | **Lowland** | | | | | | **Highland** | | | | | |
| --- | --- | --- | --- | --- | --- | --- | --- | --- | --- | --- | --- | --- |
|  | High threat | | Moderate threat | | Low threat | | High threat | | Moderate threat | | Low threat | |
| Asia | 366906.0 | 6.4% | 945863.8 | 16.6% | 4394568.1 | 77.0% | 108308.5 | 4.2% | 371597.4 | 14.5% | 2075722.1 | 81.2% |
| **National scale** |  |  |  |  |  |  |  |  |  |  |  |  |
| Afghanistan | 547.2 | 1.8% | 1551.5 | 5.1% | 28518.7 | 93.1% | 205.6 | 0.3% | 681.2 | 1.1% | 61454.9 | 98.6% |
| Armenia | 62.0 | 2.7% | 238.6 | 10.3% | 2015.6 | 87.0% | 25.5 | 0.4% | 269.2 | 4.0% | 6497.1 | 95.7% |
| Azerbaijan | 672.9 | 2.6% | 2349.3 | 9.0% | 23073.1 | 88.4% | 175.8 | 4.2% | 252.2 | 6.0% | 3806.9 | 89.9% |
| Bahrain | 0.0 | 0.0% | 2.5 | 0.9% | 270.4 | 99.1% | 0.0 | 0.0% | 0.0 | 0.0% | 0.3 | 100% |
| Bangladesh | 23731.8 | 24.1% | 28979.0 | 29.4% | 45762.0 | 46.5% | 68.2 | 12.0% | 135.0 | 23.7% | 365.7 | 64.3% |
| Bhutan | 71.0 | 14.1% | 103.1 | 20.5% | 328.7 | 65.4% | 161.3 | 3.3% | 483.7 | 9.9% | 4227.6 | 86.8% |
| Brunei | 70.9 | 9.8% | 188.3 | 25.9% | 468.2 | 64.4% | 8.6 | 12.8% | 17.2 | 25.8% | 40.9 | 61.4% |
| Cambodia | 13401.0 | 23.0% | 13262.0 | 22.8% | 31525.5 | 54.2% | 724.9 | 39.0% | 276.9 | 14.9% | 855.8 | 46.1% |
| China | 175379.4 | 8.4% | 529374.5 | 25.3% | 1391519.1 | 66.4% | 67261.3 | 4.2% | 309753.5 | 19.4% | 1222005.7 | 76.4% |
| Cyprus | 39.6 | 1.1% | 259.4 | 7.1% | 3373.4 | 91.9% | 27.9 | 4.6% | 50.5 | 8.4% | 522.3 | 87.0% |
| East Timor | 18.1 | 1.2% | 177.4 | 11.9% | 1301.2 | 86.9% | 4.6 | 1.8% | 33.2 | 13.2% | 214.3 | 85.0% |
| Georgia | 290.9 | 2.2% | 1653.4 | 12.2% | 11552.9 | 85.6% | 51.8 | 0.5% | 360.0 | 3.7% | 9336.9 | 95.8% |
| India | 26152.0 | 2.2% | 108954.0 | 9.2% | 1053320.5 | 88.6% | 2290.6 | 2.1% | 9008.1 | 8.1% | 100233.0 | 89.9% |
| Indonesia | 31921.5 | 13.5% | 46655.7 | 19.7% | 157953.5 | 66.8% | 5719.9 | 12.6% | 6356.9 | 14.0% | 33304.5 | 73.4% |
| Iran | 1418.1 | 1.4% | 6734.7 | 6.5% | 96103.6 | 92.2% | 508.4 | 0.3% | 4868.3 | 2.7% | 177727.9 | 97.1% |
| Iraq | 789.0 | 1.1% | 6311.3 | 9.1% | 62071.5 | 89.7% | 43.9 | 1.7% | 160.4 | 6.4% | 2303.8 | 91.9% |
| Israel | 308.4 | 4.1% | 1522.1 | 20.0% | 5780.2 | 75.9% | 49.9 | 8.4% | 135.7 | 22.8% | 408.6 | 68.8% |
| Japan | 1548.8 | 1.3% | 15678.5 | 12.8% | 105073.3 | 85.9% | 368.5 | 0.8% | 3620.9 | 7.8% | 42457.6 | 91.4% |
| Jordan | 313.0 | 3.9% | 901.9 | 11.3% | 6732.2 | 84.7% | 77.3 | 6.1% | 189.9 | 14.9% | 1006.1 | 79.0% |
| Kazakhstan | 4516.1 | 1.3% | 16653.3 | 4.6% | 339858.2 | 94.1% | 402.1 | 3.2% | 717.7 | 5.8% | 11268.1 | 91.0% |
| Kuwait | 17.3 | 0.7% | 82.9 | 3.4% | 2343.5 | 95.9% | 0.8 | 5.5% | 1.4 | 10.2% | 11.7 | 84.3% |
| Kyrgyzstan | 104.6 | 1.5% | 475.9 | 6.6% | 6616.6 | 91.9% | 44.5 | 0.3% | 213.4 | 1.4% | 14574.5 | 98.3% |
| Laos | 5791.3 | 16.0% | 8334.6 | 23.1% | 22030.9 | 60.9% | 2531.8 | 22.3% | 2168.8 | 19.1% | 6641.9 | 58.6% |
| Lebanon | 752.4 | 22.5% | 836.9 | 25.0% | 1759.3 | 52.5% | 409.9 | 17.6% | 594.5 | 25.5% | 1329.6 | 57.0% |
| Malaysia | 12843.3 | 29.1% | 9526.2 | 21.6% | 21816.6 | 49.4% | 1707.8 | 26.3% | 1073.9 | 16.5% | 3721.3 | 57.2% |
| Maldives | 0.0 | 0.0% | 0.0 | 0.0% | 49.8 | 100% | 0.0 | 0.0% | 0.0 | 0.0% | 0.9 | 100% |
| Mongolia | 1508.4 | 9.3% | 1699.6 | 10.5% | 13024.7 | 80.2% | 534.1 | 3.6% | 586.8 | 3.9% | 13892.8 | 92.5% |
| Myanmar | 16440.1 | 11.0% | 30843.6 | 20.6% | 102504.9 | 68.4% | 3566.9 | 11.0% | 5105.5 | 15.7% | 23805.4 | 73.3% |
| Nepal | 527.1 | 3.1% | 1754.4 | 10.3% | 14810.3 | 86.7% | 1004.0 | 2.4% | 2367.9 | 5.6% | 38540.0 | 92.0% |
| North_Korea | 580.5 | 2.5% | 2543.9 | 11.1% | 19891.8 | 86.4% | 358.2 | 2.3% | 1502.8 | 9.5% | 13928.7 | 88.2% |
| Oman | 19.1 | 0.2% | 409.8 | 4.1% | 9540.6 | 95.7% | 1.4 | 0.2% | 27.5 | 4.3% | 605.2 | 95.4% |
| Pakistan | 833.3 | 0.6% | 8107.8 | 5.9% | 129480.2 | 93.5% | 79.6 | 0.2% | 1922.9 | 4.9% | 37321.4 | 94.9% |
| Palestina | 144.8 | 5.6% | 508.8 | 19.6% | 1935.9 | 74.8% | 35.9 | 7.3% | 114.8 | 23.2% | 344.3 | 69.6% |
| Philippines | 4871.0 | 8.2% | 8822.2 | 14.9% | 45600.8 | 76.9% | 1278.5 | 11.8% | 1221.0 | 11.3% | 8318.9 | 76.9% |
| Qatar | 0.0 | 0.0% | 22.2 | 1.0% | 2311.6 | 99.0% | 0.0 | 0.0% | 0.1 | 3.4% | 1.4 | 96.6% |
| Saudi | 837.3 | 1.1% | 3470.4 | 4.5% | 73167.7 | 94.4% | 103.9 | 1.0% | 421.5 | 4.0% | 9962.6 | 95.0% |
| Singapore | 65.0 | 14.3% | 118.7 | 26.2% | 269.9 | 59.5% | 5.0 | 16.4% | 8.3 | 27.2% | 17.2 | 56.3% |
| South_Korea | 1424.3 | 3.3% | 6614.4 | 15.3% | 35172.0 | 81.4% | 308.4 | 1.3% | 1770.9 | 7.3% | 22052.4 | 91.4% |
| Sri_Lanka | 1571.0 | 5.6% | 4141.4 | 14.8% | 22336.0 | 79.6% | 100.3 | 3.5% | 181.9 | 6.4% | 2577.4 | 90.1% |
| Syria | 326.1 | 0.8% | 2742.4 | 6.6% | 38767.1 | 92.7% | 69.8 | 2.2% | 316.1 | 10.1% | 2747.1 | 87.7% |
| Tajikistan | 92.4 | 1.0% | 714.7 | 7.5% | 8672.8 | 91.5% | 47.6 | 0.7% | 182.8 | 2.9% | 6169.7 | 96.4% |
| Thailand | 14070.8 | 8.2% | 30721.4 | 17.8% | 127341.3 | 74.0% | 4692.9 | 25.9% | 2674.4 | 14.7% | 10782.2 | 59.4% |
| Turkey | 1977.7 | 1.1% | 10358.1 | 6.0% | 160738.0 | 92.9% | 996.1 | 0.7% | 4353.6 | 2.9% | 143092.2 | 96.4% |
| Turkmenistan | 81.4 | 0.2% | 1613.4 | 4.9% | 31068.8 | 94.8% | 2.4 | 0.6% | 22.4 | 5.8% | 363.6 | 93.6% |
| United Arab Emirates | 20.3 | 0.2% | 392.6 | 3.3% | 11463.7 | 96.5% | 1.7 | 0.6% | 13.1 | 4.9% | 253.0 | 94.5% |
| Uzbekistan | 264.5 | 0.4% | 4742.9 | 7.6% | 57522.1 | 92.0% | 67.5 | 1.4% | 298.8 | 6.4% | 4329.6 | 92.2% |
| Vietnam | 20076.7 | 20.4% | 23741.2 | 24.1% | 54681.4 | 55.5% | 11853.3 | 32.4% | 6633.3 | 18.2% | 18060.1 | 49.4% |
| Yemen | 413.6 | 2.9% | 972.9 | 6.7% | 13047.9 | 90.4% | 330.2 | 2.2% | 448.9 | 3.0% | 14239.3 | 94.8% |
| **Hotspot scale** |  |  |  |  |  |  |  |  |  |  |  |  |
| Asian terrestrial PAs | 6186.1 | 10.0% | 8271.4 | 13.3% | 47593.5 | 76.7% | 2467.5 | 8.3% | 3218.8 | 10.8% | 24089.9 | 80.9% |

**Supplementary Table 5. Information of terrestrial PAs** **established before 2000 in Asia (after excluding point features and merging overlapped features).**

|  | **Lowland PAs** | | **Highland PAs** | | **Total PAs** | |
| --- | --- | --- | --- | --- | --- | --- |
|  | Area (km^2^) | Proportion (%) | Area (km^2^) | Proportion (%) | Area (km^2^) | Number |
| **Asia** | 421635.2 | 43.2 | 553913.8 | 56.8 | 975549.0 | 6502 |

**Supplementary Table 6. The average values of threat degrees in different countries’ PAs (only 26 countries’ PAs have regions with threat degrees).**

| Terrestrial PAs in different countries | Overall PAs average (%) | Lowland PAs  average (%) | Highland PAs average (%) | Difference value (%) (highland-lowland) |
| --- | --- | --- | --- | --- |
| Afghanistan | 1.7 | 1.7 | 0.0 | -1.7 |
| Azerbaijan | 7.1 | 7.3 | 6.4 | -0.9 |
| Bangladesh | 11.2 | 11.2 | 11.6 | 0.4 |
| Bhutan | 6.1 | 9.5 | 5.6 | -3.9 |
| Brunei | 8.8 | 8.7 | 9.6 | 0.9 |
| Cambodia | 14.4 | 14.5 | 13.8 | -0.7 |
| China | 10.8 | 11.5 | 10.0 | -1.5 |
| East Timor | 7.6 | 7.7 | 7.4 | -0.2 |
| India | 7.7 | 7.7 | 7.6 | -0.1 |
| Indonesia | 12.9 | 14.4 | 11.1 | -3.3 |
| Iran | 5.6 | 6.4 | 5.3 | -1.1 |
| Iraq | 3.3 | 2.7 | 3.6 | 1.0 |
| Japan | 7.0 | 7.4 | 6.4 | -1.0 |
| Laos | 11.9 | 12.0 | 11.7 | -0.3 |
| Malaysia | 11.1 | 11.5 | 10.0 | -1.5 |
| Mongolia | 12.9 | 14.4 | 9.5 | -4.9 |
| Myanmar | 9.5 | 9.2 | 11.1 | 1.9 |
| Nepal | 6.6 | 7.0 | 6.5 | -0.6 |
| North_Korea | 4.7 | 5.8 | 4.6 | -1.2 |
| Pakistan | 6.0 | 6.0 | 6.1 | 0.1 |
| Philippines | 9.0 | 9.1 | 8.6 | -0.4 |
| Singapore | 8.3 | 8.4 | 7.6 | -0.9 |
| South_Korea | 6.4 | 6.9 | 5.8 | -1.1 |
| Sri_Lanka | 10.4 | 10.6 | 8.5 | -2.1 |
| Thailand | 12.9 | 12.1 | 14.3 | 2.1 |
| Vietnam | 11.6 | 10.6 | 13.7 | 3.1 |

**Supplementary Table 7. Proportions (%) and areas (km^2^) of three threat degrees (i.e., high, moderate and low) in different countries’ PAs (only 26 countries’ PAs have regions with threat degrees).**

| Terrestrial PAs in different countries | High threat | | Moderate threat | | Low threat | |
| --- | --- | --- | --- | --- | --- | --- |
| Afghanistan | 0.0 | 0.0% | 0.0 | 0.0% | 0.3 | 100% |
| Azerbaijan | 0.0 | 0.0% | 0.2 | 3.9% | 3.7 | 96.1% |
| Bangladesh | 76.1 | 13.5% | 148.8 | 26.4% | 338.2 | 60.1% |
| Bhutan | 8.2 | 2.3% | 14.6 | 4.1% | 332.9 | 93.6% |
| Brunei | 0.0 | 0.0% | 2.8 | 18.5% | 12.3 | 81.5% |
| Cambodia | 1404.0 | 29.3% | 1097.0 | 22.9% | 2291.5 | 47.8% |
| China | 1466.9 | 12.4% | 2783.8 | 23.6% | 7538.7 | 63.9% |
| East Timor | 0.7 | 2.9% | 3.3 | 14.3% | 19.1 | 82.9% |
| India | 36.4 | 5.2% | 93.3 | 13.3% | 572.9 | 81.5% |
| Indonesia | 162.7 | 21.5% | 136.4 | 18.0% | 458.6 | 60.5% |
| Iran | 27.4 | 0.4% | 271.4 | 3.8% | 6898.7 | 95.8% |
| Iraq | 0.0 | 0.0% | 0.0 | 0.0% | 0.2 | 100% |
| Japan | 194.6 | 1.1% | 1772.6 | 9.9% | 15850.7 | 89.0% |
| Laos | 412.8 | 19.2% | 435.3 | 20.2% | 1306.9 | 60.6% |
| Malaysia | 38.0 | 16.5% | 44.4 | 19.3% | 147.6 | 64.2% |
| Mongolia | 285.6 | 28.1% | 179.7 | 17.7% | 552.5 | 54.3% |
| Myanmar | 70.0 | 9.4% | 113.6 | 15.2% | 562.7 | 75.4% |
| Nepal | 42.1 | 1.8% | 146.7 | 6.2% | 2171.5 | 92.0% |
| North_Korea | 0.0 | 0.0% | 0.0 | 0.0% | 0.6 | 100% |
| Pakistan | 61.7 | 0.4% | 724.2 | 4.8% | 14224.2 | 94.8% |
| Philippines | 419.8 | 8.9% | 524.7 | 11.1% | 3781.9 | 80.0% |
| Singapore | 0.0 | 0.0% | 1.8 | 20.6% | 7.0 | 79.4% |
| South_Korea | 41.5 | 1.2% | 230.9 | 6.4% | 3314.8 | 92.4% |
| Sri_Lanka | 725.4 | 16.3% | 761.5 | 17.1% | 2971.5 | 66.6% |
| Thailand | 2682.6 | 25.1% | 1452.1 | 13.6% | 6558.9 | 61.3% |
| Vietnam | 497.1 | 17.7% | 551.3 | 19.6% | 1765.3 | 62.7% |

**Supplementary Table 8. Proportions (%) and areas (km^2^) of three threat degrees (i.e., high, moderate and low) in lowland and highland PAs in different countries (only 26 countries’ PAs have regions with threat degrees).**

|  | **Lowland PAs** | | | | | | **Highland PAs** | | | | | |
| --- | --- | --- | --- | --- | --- | --- | --- | --- | --- | --- | --- | --- |
| Terrestrial PAs in different countries | High threat | | Moderate threat | | Low threat | | High threat | | Moderate threat | | Low threat | |
| Afghanistan | 0.0 | 0.0% | 0.0 | 0.0% | 0.3 | 100% | 0.0 | 0.0% | 0.0 | 0.0% | 0.0 | 0.0% |
| Azerbaijan | 0.0 | 0.0% | 0.2 | 5.9% | 3.2 | 94.1% | 0.0 | 0.0% | 0.0 | 0.0% | 0.5 | 100% |
| Bangladesh | 73.5 | 13.5% | 143.8 | 26.4% | 328.4 | 60.2% | 2.6 | 14.9% | 5.0 | 28.6% | 9.9 | 56.6% |
| Bhutan | 6.0 | 12.6% | 6.0 | 12.6% | 35.7 | 74.8% | 2.1 | 0.7% | 8.7 | 2.8% | 297.2 | 96.5% |
| Brunei | 0.0 | 0.0% | 2.4 | 17.9% | 11.0 | 82.1% | 0.0 | 0.0% | 0.4 | 23.5% | 1.3 | 76.5% |
| Cambodia | 1343.1 | 29.4% | 1060.6 | 23.2% | 2169.6 | 47.4% | 61.0 | 27.8% | 36.4 | 16.6% | 121.9 | 55.6% |
| China | 1020.2 | 16.7% | 1497.6 | 24.5% | 3583.5 | 58.7% | 446.7 | 7.9% | 1286.1 | 22.6% | 3955.2 | 69.5% |
| East Timor | 0.6 | 5.0% | 1.3 | 10.7% | 10.2 | 84.3% | 0.1 | 0.9% | 2.0 | 18.2% | 8.9 | 80.9% |
| India | 34.4 | 5.0% | 91.9 | 13.4% | 561.6 | 81.6% | 2.0 | 13.6% | 1.4 | 9.5% | 11.3 | 76.9% |
| Indonesia | 102.6 | 25.4% | 80.6 | 19.9% | 221.0 | 54.7% | 60.0 | 17.0% | 55.8 | 15.8% | 237.6 | 67.2% |
| Iran | 19.3 | 0.8% | 169.2 | 7.3% | 2116.7 | 91.8% | 8.0 | 0.2% | 102.1 | 2.1% | 4782.0 | 97.7% |
| Iraq | 0.0 | 0.0% | 0.0 | 0.0% | 0.1 | 100% | 0.0 | 0.0% | 0.0 | 0.0% | 0.1 | 100% |
| Japan | 152.6 | 1.3% | 1340.8 | 11.7% | 9924.0 | 86.9% | 42.0 | 0.7% | 431.9 | 6.7% | 5926.7 | 92.6% |
| Laos | 279.0 | 19.0% | 315.8 | 21.5% | 872.0 | 59.4% | 133.8 | 19.4% | 119.4 | 17.4% | 434.9 | 63.2% |
| Malaysia | 29.4 | 17.1% | 36.3 | 21.1% | 106.7 | 61.9% | 8.6 | 14.9% | 8.1 | 14.1% | 40.9 | 71.0% |
| Mongolia | 233.7 | 33.4% | 147.8 | 21.1% | 317.5 | 45.4% | 51.9 | 16.3% | 31.9 | 10.0% | 235.1 | 73.7% |
| Myanmar | 51.6 | 8.3% | 87.6 | 14.1% | 480.7 | 77.5% | 18.4 | 14.6% | 25.9 | 20.5% | 82.0 | 64.9% |
| Nepal | 10.6 | 1.4% | 62.4 | 8.2% | 686.8 | 90.4% | 31.5 | 2.0% | 84.3 | 5.3% | 1484.7 | 92.8% |
| North_Korea | 0.0 | 0.0% | 0.0 | 0.0% | 0.1 | 100% | 0.0 | 0.0% | 0.0 | 0.0% | 0.5 | 100% |
| Pakistan | 57.2 | 0.4% | 641.6 | 4.7% | 13036.8 | 94.9% | 4.5 | 0.4% | 82.6 | 6.5% | 1187.4 | 93.2% |
| Philippines | 320.2 | 8.7% | 429.5 | 11.7% | 2931.5 | 79.6% | 99.6 | 9.5% | 95.2 | 9.1% | 850.4 | 81.4% |
| Singapore | 0.0 | 0.0% | 1.5 | 20.8% | 5.7 | 79.2% | 0.0 | 0.0% | 0.4 | 23.5% | 1.3 | 76.5% |
| South_Korea | 32.1 | 1.7% | 161.3 | 8.3% | 1749.8 | 90.0% | 9.3 | 0.6% | 69.6 | 4.2% | 1565.0 | 95.2% |
| Sri Lanka | 682.5 | 16.7% | 740.6 | 18.2% | 2656.4 | 65.1% | 42.9 | 11.3% | 20.9 | 5.5% | 315.1 | 83.2% |
| Thailand | 1483.7 | 21.4% | 948.9 | 13.7% | 4503.3 | 64.9% | 1199.0 | 31.9% | 503.2 | 13.4 % | 2055.6 | 54.7% |
| Vietnam | 253.7 | 13.8% | 303.7 | 16.5% | 1280.8 | 69.7% | 243.5 | 25.0% | 247.6 | 25.4% | 484.5 | 49.7% |

**Supplementary Table 9. The average correlation degree tests of integration index with three density map.**

| Pearson coefficient | Density map of patch number | Density map of patch area | Density map of number of threatened species involved in patches | Integration index derived-threat degree |
| --- | --- | --- | --- | --- |
| Density map of patch number | 1 | 0.05^*^ | 0.07^**^ | 0.68^**^ |
| Density map of patch area | 0.05^*^ | 1 | 0.04 | 0.61^**^ |
| Density map of number of threatened species involved in patches | 0.07^**^ | 0.04 | 1 | 0.29^**^ |
| Integration index derived-threat degree | 0.68^**^ | 0.61^**^ | 0.29^**^ | 1 |
| **Average correlation degree** | **0.27** | **0.23** | **0.13** | **0.53** |

***** Correlation is signifcant at the 0.05 level (2-tailed)

****** Correlation is signifcant at the 0.01 level (2-tailed)

**Supplementary Table 10.** **The total number of threatened species in Asia and 48 Asian countries, including threatened species in lowlands and highlands.** **Threatened species comprise the species assessed as Critically Endangered (CR), Endangered (EN), and Vulnerable (VU) Red List categories.**

| **Continental scale** | Threatened species types | Lowland | Highland | Total |
| --- | --- | --- | --- | --- |
| Asia | mammals | 419 | 436 | 438 |
|  | amphibians | 460 | 503 | 503 |
|  | reptiles | 344 | 349 | 351 |
|  | birds | 438 | 440 | 442 |
|  | plant | 176 | 257 | 269 |
|  | **Total** | **1837** | **1985** | **2003** |
| **National scale** |  |  |  |  |
| Kazakhstan | mammals | 8 | 9 | 9 |
|  | amphibians | 1 | 1 | 1 |
|  | reptiles | 0 | 0 | 0 |
|  | birds | 23 | 23 | 23 |
|  | plant | 0 | 0 | 0 |
|  | **Total** | **32** | **33** | **33** |
| Kyrgyzstan | mammals | 3 | 4 | 4 |
|  | amphibians | 0 | 0 | 0 |
|  | reptiles | 4 | 4 | 4 |
|  | birds | 15 | 15 | 15 |
|  | plant | 0 | 0 | 0 |
|  | **Total** | **22** | **23** | **23** |
| Tajikistan | mammals | 5 | 5 | 5 |
|  | amphibians | 0 | 0 | 0 |
|  | reptiles | 5 | 5 | 5 |
|  | birds | 15 | 15 | 15 |
|  | plant | 0 | 0 | 0 |
|  | **Total** | **25** | **25** | **25** |
| Turkmenistan | mammals | 4 | 4 | 4 |
|  | amphibians | 0 | 0 | 0 |
|  | reptiles | 5 | 5 | 5 |
|  | birds | 17 | 17 | 17 |
|  | plant | 0 | 0 | 0 |
|  | **Total** | **26** | **26** | **26** |
| Uzbekistan | mammals | 6 | 7 | 7 |
|  | amphibians | 0 | 0 | 0 |
|  | reptiles | 8 | 8 | 8 |
|  | birds | 15 | 15 | 15 |
|  | plant | 0 | 0 | 0 |
|  | **Total** | **29** | **30** | **30** |
| Bangladesh | mammals | 24 | 23 | 24 |
|  | amphibians | 2 | 2 | 2 |
|  | reptiles | 28 | 27 | 28 |
|  | birds | 40 | 39 | 40 |
|  | plant | 2 | 2 | 2 |
|  | **Total** | **96** | **93** | **96** |
| Bhutan | mammals | 24 | 25 | 25 |
|  | amphibians | 1 | 1 | 1 |
|  | reptiles | 19 | 19 | 19 |
|  | birds | 29 | 29 | 30 |
|  | plant | 1 | 1 | 1 |
|  | **Total** | **74** | **75** | **76** |
| India | mammals | 73 | 77 | 78 |
|  | amphibians | 71 | 75 | 75 |
|  | reptiles | 64 | 66 | 66 |
|  | birds | 92 | 91 | 92 |
|  | plant | 6 | 8 | 8 |
|  | **Total** | **306** | **317** | **319** |
| Pakistan | mammals | 10 | 14 | 14 |
|  | amphibians | 0 | 0 | 0 |
|  | reptiles | 12 | 12 | 12 |
|  | birds | 32 | 33 | 33 |
|  | plant | 0 | 0 | 0 |
|  | **Total** | **54** | **59** | **59** |
| Maldives | mammals | 0 | 0 | 0 |
|  | amphibians | 0 | 0 | 0 |
|  | reptiles | 1 | 4 | 4 |
|  | birds | 4 | 4 | 4 |
|  | plant | 0 | 0 | 0 |
|  | **Total** | **5** | **8** | **8** |
| Nepal | mammals | 20 | 21 | 21 |
|  | amphibians | 3 | 3 | 3 |
|  | reptiles | 16 | 16 | 16 |
|  | birds | 39 | 39 | 39 |
|  | plant | 1 | 1 | 1 |
|  | **Total** | **79** | **80** | **80** |
| Sri Lanka | mammals | 22 | 22 | 22 |
|  | amphibians | 71 | 75 | 75 |
|  | reptiles | 23 | 22 | 23 |
|  | birds | 13 | 13 | 13 |
|  | plant | 0 | 0 | 0 |
|  | **Total** | **129** | **132** | **133** |
| China | mammals | 65 | 76 | 76 |
|  | amphibians | 127 | 152 | 152 |
|  | reptiles | 57 | 58 | 59 |
|  | birds | 108 | 111 | 112 |
|  | plant | 94 | 107 | 112 |
|  | **Total** | **451** | **504** | **511** |
| Japan | mammals | 20 | 20 | 20 |
|  | amphibians | 43 | 43 | 43 |
|  | reptiles | 22 | 22 | 22 |
|  | birds | 45 | 44 | 45 |
|  | plant | 4 | 5 | 4 |
|  | **Total** | 134 | 134 | 134 |
| South Korea | mammals | 4 | 4 | 4 |
|  | amphibians | 5 | 5 | 5 |
|  | reptiles | 4 | 4 | 4 |
|  | birds | 28 | 26 | 28 |
|  | plant | 1 | 2 | 2 |
|  | **Total** | 42 | 41 | 43 |
| Mongolia | mammals | 9 | 10 | 10 |
|  | amphibians | 0 | 0 | 0 |
|  | reptiles | 0 | 0 | 0 |
|  | birds | 22 | 24 | 24 |
|  | plant | 0 | 0 | 0 |
|  | **Total** | 31 | 34 | 34 |
| North Korea | mammals | 4 | 4 | 4 |
|  | amphibians | 2 | 2 | 2 |
|  | reptiles | 4 | 4 | 4 |
|  | birds | 24 | 22 | 24 |
|  | plant | 0 | 0 | 0 |
|  | **Total** | 34 | 32 | 34 |
| Brunei | mammals | 34 | 34 | 34 |
|  | amphibians | 3 | 3 | 3 |
|  | reptiles | 14 | 14 | 14 |
|  | birds | 36 | 36 | 36 |
|  | plant | 4 | 4 | 4 |
|  | **Total** | 91 | 91 | 91 |
| Cambodia | mammals | 30 | 30 | 30 |
|  | amphibians | 11 | 11 | 11 |
|  | reptiles | 25 | 25 | 25 |
|  | birds | 32 | 32 | 32 |
|  | plant | 7 | 7 | 7 |
|  | **Total** | 105 | 105 | 105 |
| East Timor | mammals | 5 | 5 | 5 |
|  | amphibians | 0 | 0 | 0 |
|  | reptiles | 7 | 7 | 7 |
|  | birds | 8 | 8 | 8 |
|  | plant | 1 | 1 | 1 |
|  | **Total** | 21 | 21 | 21 |
| Indonesia | mammals | 197 | 201 | 202 |
|  | amphibians | 30 | 33 | 33 |
|  | reptiles | 43 | 43 | 43 |
|  | birds | 169 | 169 | 169 |
|  | plant | 10 | 17 | 18 |
|  | **Total** | 449 | 463 | 465 |
| Laos | mammals | 66 | 66 | 66 |
|  | amphibians | 15 | 15 | 15 |
|  | reptiles | 32 | 32 | 32 |
|  | birds | 36 | 36 | 36 |
|  | plant | 9 | 8 | 9 |
|  | **Total** | 158 | 157 | 158 |
| Malaysia | mammals | 66 | 66 | 66 |
|  | amphibians | 30 | 35 | 35 |
|  | reptiles | 56 | 58 | 58 |
|  | birds | 64 | 64 | 64 |
|  | plant | 17 | 18 | 18 |
|  | **Total** | 233 | 241 | 241 |
| Myanmar | mammals | 46 | 49 | 49 |
|  | amphibians | 8 | 8 | 8 |
|  | reptiles | 37 | 37 | 37 |
|  | birds | 61 | 61 | 61 |
|  | plant | 16 | 16 | 17 |
|  | **Total** | 168 | 171 | 172 |
| Philippines | mammals | 36 | 36 | 36 |
|  | amphibians | 27 | 27 | 27 |
|  | reptiles | 40 | 40 | 40 |
|  | birds | 90 | 89 | 90 |
|  | plant | 1 | 1 | 1 |
|  | **Total** | 194 | 193 | 194 |
| Singapore | mammals | 6 | 6 | 6 |
|  | amphibians | 64 | 64 | 64 |
|  | reptiles | 15 | 15 | 15 |
|  | birds | 31 | 29 | 31 |
|  | plant | 2 | 0 | 2 |
|  | **Total** | 118 | 114 | 118 |
| Thailand | mammals | 56 | 55 | 111 |
|  | amphibians | 11 | 11 | 11 |
|  | reptiles | 39 | 39 | 39 |
|  | birds | 64 | 64 | 64 |
|  | plant | 9 | 12 | 12 |
|  | **Total** | 179 | 181 | 237 |
| Vietnam | mammals | 54 | 54 | 54 |
|  | amphibians | 46 | 52 | 52 |
|  | reptiles | 69 | 69 | 69 |
|  | birds | 49 | 49 | 49 |
|  | plant | 39 | 41 | 41 |
|  | **Total** | 257 | 265 | 265 |
| Afghanistan | mammals | 7 | 8 | 8 |
|  | amphibians | 0 | 1 | 1 |
|  | reptiles | 2 | 1 | 2 |
|  | birds | 12 | 13 | 13 |
|  | plant | 0 | 0 | 0 |
|  | **Total** | 21 | 23 | 24 |
| Armenia | mammals | 3 | 5 | 5 |
|  | amphibians | 1 | 1 | 1 |
|  | reptiles | 3 | 5 | 5 |
|  | birds | 15 | 15 | 15 |
|  | plant | 0 | 0 | 0 |
|  | **Total** | 22 | 26 | 26 |
| Azerbaijan | mammals | 6 | 6 | 6 |
|  | amphibians | 1 | 1 | 1 |
|  | reptiles | 6 | 7 | 7 |
|  | birds | 15 | 15 | 15 |
|  | plant | 0 | 0 | 0 |
|  | **Total** | 28 | 29 | 29 |
| Bahrain | mammals | 1 | 1 | 1 |
|  | amphibians | 0 | 0 | 0 |
|  | reptiles | 6 | 6 | 6 |
|  | birds | 7 | 6 | 7 |
|  | plant | 0 | 0 | 0 |
|  | **Total** | 14 | 13 | 14 |
| Cyprus | mammals | 4 | 4 | 4 |
|  | amphibians | 0 | 0 | 0 |
|  | reptiles | 6 | 6 | 6 |
|  | birds | 8 | 8 | 8 |
|  | plant | 0 | 0 | 0 |
|  | **Total** | 18 | 18 | 18 |
| Georgia | mammals | 7 | 7 | 7 |
|  | amphibians | 1 | 1 | 1 |
|  | reptiles | 6 | 6 | 6 |
|  | birds | 15 | 15 | 15 |
|  | plant | 1 | 1 | 1 |
|  | **Total** | **30** | **30** | **30** |
| Iran | mammals | 11 | 11 | 12 |
|  | amphibians | 4 | 5 | 5 |
|  | reptiles | 12 | 13 | 13 |
|  | birds | 23 | 23 | 23 |
|  | plant | 1 | 4 | 4 |
|  | **Total** | **51** | **56** | **57** |
| Iraq | mammals | 6 | 6 | 6 |
|  | amphibians | 1 | 1 | 1 |
|  | reptiles | 5 | 2 | 5 |
|  | birds | 17 | 16 | 17 |
|  | plant | 0 | 0 | 0 |
|  | **Total** | **29** | **25** | **29** |
| Israel | mammals | 11 | 11 | 11 |
|  | amphibians | 1 | 1 | 1 |
|  | reptiles | 14 | 14 | 15 |
|  | birds | 16 | 16 | 16 |
|  | plant | 0 | 0 | 0 |
|  | **Total** | **42** | **42** | **43** |
| Jordan | mammals | 8 | 7 | 8 |
|  | amphibians | 0 | 0 | 0 |
|  | reptiles | 8 | 5 | 8 |
|  | birds | 14 | 14 | 14 |
|  | plant | 0 | 0 | 0 |
|  | **Total** | **30** | **26** | **30** |
| Kuwait | mammals | 1 | 1 | 1 |
|  | amphibians | 0 | 0 | 0 |
|  | reptiles | 5 | 2 | 5 |
|  | birds | 11 | 10 | 11 |
|  | plant | 0 | 0 | 0 |
|  | **Total** | **17** | **13** | **17** |
| Lebanon | mammals | 6 | 6 | 6 |
|  | amphibians | 0 | 0 | 0 |
|  | reptiles | 11 | 9 | 11 |
|  | birds | 13 | 13 | 13 |
|  | plant | 0 | 0 | 0 |
|  | **Total** | **30** | **28** | **30** |
| Oman | mammals | 6 | 6 | 6 |
|  | amphibians | 0 | 0 | 0 |
|  | reptiles | 7 | 7 | 7 |
|  | birds | 13 | 13 | 13 |
|  | plant | 0 | 0 | 0 |
|  | **Total** | **26** | **26** | **26** |
| Palestine | mammals | 8 | 7 | 8 |
|  | amphibians | 0 | 0 | 0 |
|  | reptiles | 15 | 14 | 15 |
|  | birds | 9 | 7 | 9 |
|  | plant | 0 | 0 | 0 |
|  | **Total** | **32** | **28** | **32** |
| Qatar | mammals | 1 | 1 | 1 |
|  | amphibians | 0 | 0 | 0 |
|  | reptiles | 4 | 4 | 4 |
|  | birds | 9 | 9 | 9 |
|  | plant | 0 | 0 | 0 |
|  | **Total** | **14** | **14** | **14** |
| Saudi Arabia | mammals | 6 | 6 | 6 |
|  | amphibians | 0 | 0 | 0 |
|  | reptiles | 5 | 5 | 5 |
|  | birds | 18 | 18 | 18 |
|  | plant | 0 | 0 | 0 |
|  | **Total** | **29** | **29** | **29** |
| Syria | mammals | 9 | 9 | 9 |
|  | amphibians | 0 | 0 | 0 |
|  | reptiles | 9 | 11 | 11 |
|  | birds | 17 | 17 | 17 |
|  | plant | 0 | 0 | 0 |
|  | **Total** | **35** | **37** | **37** |
| Turkey | mammals | 13 | 13 | 13 |
|  | amphibians | 9 | 10 | 10 |
|  | reptiles | 20 | 21 | 22 |
|  | birds | 18 | 16 | 18 |
|  | plant | 1 | 2 | 2 |
|  | **Total** | **61** | **62** | **65** |
| United Arab Emirates | mammals | 5 | 5 | 5 |
|  | amphibians | 0 | 0 | 0 |
|  | reptiles | 5 | 5 | 5 |
|  | birds | 13 | 12 | 13 |
|  | plant | 0 | 0 | 0 |
|  | **Total** | **23** | **22** | **23** |
| Yemen | mammals | 7 | 7 | 7 |
|  | amphibians | 1 | 1 | 1 |
|  | reptiles | 7 | 7 | 7 |
|  | birds | 16 | 16 | 16 |
|  | plant | 0 | 0 | 0 |
|  | **Total** | **31** | **31** | **31** |

**Supplementary Table 11. Criteria used to determine lowland and highland in Asia.**

| Categories | Broad physiographic features | Criteria of elevation and slope classes |
| --- | --- | --- |
| Lowland | Plains and terraces | elevation 0-1000m with slope 0-15° elevation 1000-1200m with slope 0-8° |
| Highland | Hills | elevation 0-1000 m with slope >15° elevation 1000-1200 m with slope >8° elevation 1200-1500 with slope 0-3° |
|  | Mountains | elevation 1200-1500 with slope >3° and elevation >1500m |

.
